# Species conservation profiles of cave-dwelling arthropods from Azores, Portugal

**DOI:** 10.3897/BDJ.7.e32530

**Published:** 2019-04-24

**Authors:** Paulo Alexandre Vieira Borges, Lucas Lamelas-Lopez, Isabel R. Amorim, Anja Danielczak, Mário Boieiro, Carla Rego, Sophie Wallon, Rui Nunes, Pedro Cardoso, Axel Hochkirch

**Affiliations:** 1 CE3C – Centre for Ecology, Evolution and Environmental Changes / Azorean Biodiversity Group and Universidade dos Açores, Dep. de Ciências e Engenharia do Ambiente, Angra do Heroísmo, Açores, Portugal CE3C – Centre for Ecology, Evolution and Environmental Changes / Azorean Biodiversity Group and Universidade dos Açores, Dep. de Ciências e Engenharia do Ambiente Angra do Heroísmo, Açores Portugal; 2 IUCN SSC Mid-Atlantic Islands Specialist Group, Angra do Heroísmo, Açores, Portugal IUCN SSC Mid-Atlantic Islands Specialist Group Angra do Heroísmo, Açores Portugal; 3 Trier University, Department of Biogeography, D-54296 Trier, Germany Trier University, Department of Biogeography D-54296 Trier Germany; 4 CE3C – Centre for Ecology, Evolution and Environmental Changes / Azorean Biodiversity Group and Universidade dos Açores, Angra do Heroísmo, Açores, Portugal CE3C – Centre for Ecology, Evolution and Environmental Changes / Azorean Biodiversity Group and Universidade dos Açores Angra do Heroísmo, Açores Portugal; 5 LIBRe - Laboratory for Integrative Biodiversity Research, Finnish Museum of Natural History, University of Helsinki, Helsinki, Finland LIBRe - Laboratory for Integrative Biodiversity Research, Finnish Museum of Natural History, University of Helsinki Helsinki Finland; 6 IUCN SSC Spider & Scorpion Specialist Group, Helsinki, Finland IUCN SSC Spider & Scorpion Specialist Group Helsinki Finland

**Keywords:** Arthropoda, extinction risk, cave-adapted species, islands, IUCN, troglobionts, Red List

## Abstract

**Background:**

Azorean volcanic cave biodiversity is under considerable pressure due to ongoing threats of pollution, land use change, touristic activities or climate change. In this contribution, we present the IUCN Red List profiles of 15 cave-adapted arthropod species, endemic to the Azorean archipelago, including species belonging to the speciose genus *Trechus* (Carabidae), which is represented in Azores by seven species. The objective of this paper is to assess all endemic Azorean cave-adapted species and advise on possible future research and conservation actions critical for the long-term survival of the most endangered species.

**New information:**

Most species have a restricted distribution (i.e. occur in one or two caves), very small extent of occurrence (EOO) and a small area of occupancy (AOO). A continuing decline in the number of mature individuals is inferred from the ongoing cave habitat degradation. The two troglobitic species of the homopteran genus *Cixius* are in great danger of extinction due to major land-use changes in epigean habitats above their known localities. We suggest, as future measures of conservation, the regular monitoring of the species (every five years), the creation of additional protected caves, the limitation of several aggressive activities around the caves (e.g. decreasing pasture intensification) and in some cases the creation of fences in the entrance of the most important caves.

## Introduction

Three archipelagos of Macaronesia (Azores, Madeira, Canaries) have unique arthropod cave biological diversity (Oromí and Izquierdo 1994, Reboleira et al. 2011, Borges et al. 2012). The dynamic volcanic activity and isolation were important drivers of species richness, as well as endemism in these archipelagos (Borges and Hortal 2009). A major fraction of these species are obligate cave-adapted species, i.e. troglobionts and tend to have very restricted distributions (Borges et al. 2012). A total of 272 cavities were recently listed in a database of the Azorean caves (Pereira et al. 2015), but for only 42 (37 lava-tubes and 5 volcanic pits), there is adequate knowledge on their fauna (Borges et al. 2012).

The currently known diversity of Azorean troglobiont arthropods is composed of 17 described species and subspecies and four additional undescribed species (Borges and Oromí 1994, Borges et al. 2012), all endemic to the archipelago. However, for some species (e.g. some of the Collembola species and the crustacean Amphipod *Macarorchestia martini*; see Wildish 2014 that mentions it as potentially trogloxene or driftwood specialist), the troglobiont status needs to be confirmed with additional research on its biology. Eight arthropod orders are represented, including the arachnids Pseudoscorpiones (two species) and Araneae (two species), the crustacean Amphipoda (one species) and Isopoda (one undescribed species), the centipede Lithobiomorpha (one subspecies), Collembola (two species, one of them undescribed) and, finally, insects, with Hemiptera (two described and two undescribed species) and Coleoptera (eight species) (Borges and Oromí 1994).

After a previous assessment of the cave spider *Turinyphia cavernicola* (Borges et al. 2016), in this contribution we present the IUCN Red List profiles of 15 additional cave-adapted arthropod species endemic to the Azores, leaving out the centipede subspecies *Lithobius obscurus azoreae*. The Hemiptera (two species) and Coleoptera (eight species) assessments have already been included in the IUCN Red List of Threatened Species and have a Red List Category assigned: CR (five species), EN (two species) and VU (three species) (see also https://www.iucnredlist.org and http://www.maiisg.com/).

## Methods

To create the IUCN Red List profiles, we followed the standard procedures as in Borges et al. (2016), Borges et al. (2017) and Borges et al. (2018): i) the original species descriptions were investigated to learn about the habitats and ecology of the species; ii) all the most recent literature was also investigated to obtain information about synonyms and critical information for the taxonomic notes; iii) for the calculation of AOO and EOO, we consulted the Azorean Biodiversity Portal and downloaded CSV files with the distribution of each species; iv) images of the species were also obtained from the repository available in the Azorean Biodiversity Portal, the most important source of information for Azores biodiversity (see Borges et al. 2010a).

Prior to the calculation of area of occupancy (AOO) and extent of occurrence (EOO), the 500 m x 500 m cells obtained from Azorean Biodiversity Portal were filtered to consider only the cells with high level of precision as defined by: 1 – very precise location, usually point UTM data; 2 – literature location not exceeding 25 km^2^. The centroid for each cell was calculated to obtain the distribution points for each species. The calculation of AOO and EOO was performed using the Geospatial Conservation Assessment Tool (GeoCAT) and using an approximation to the standard IUCN 2 km × 2 km cells (4 km^2^). Final maps with species distributions were produced using the IUCN standards with Google Earth (kmz files).

Critical information on species threats and conservation were mostly obtained from Borges et al. (2004), Borges et al. (2007) and Borges et al. (2012).

## Species Conservation Profiles

### Pseudoblothrus oromii

#### Species information

Scientific name: Pseudoblothrus oromii

Species authority: Mahnert,1990

Kingdom: Animalia

Phylum: Arthropoda

Class: Arachnida

Order: Pseudoscorpion

Family: Syarinidae

Figure(s) or Photo(s): 

Region for assessment: Global

#### Editor & Reviewers

##### Reviewers

Reviewers: Dinarte Teixeira

##### Editor

Editor: Axel Hochkirch

#### Reviewers

Reviewers: Dinarte Teixeira

#### Editor

Editor: Axel Hochkirch

#### Geographic range

Biogeographic realm: Palearctic

Countries: Portugal

Map of records (Google Earth): Suppl. material 3

Basis of EOO and AOO: Known habitat extent

Basis (narrative): The extent of occurrence (EOO) is 4 km² and the maximum estimated area of occupancy (AOO) is 4 km².

**Figure 1.**
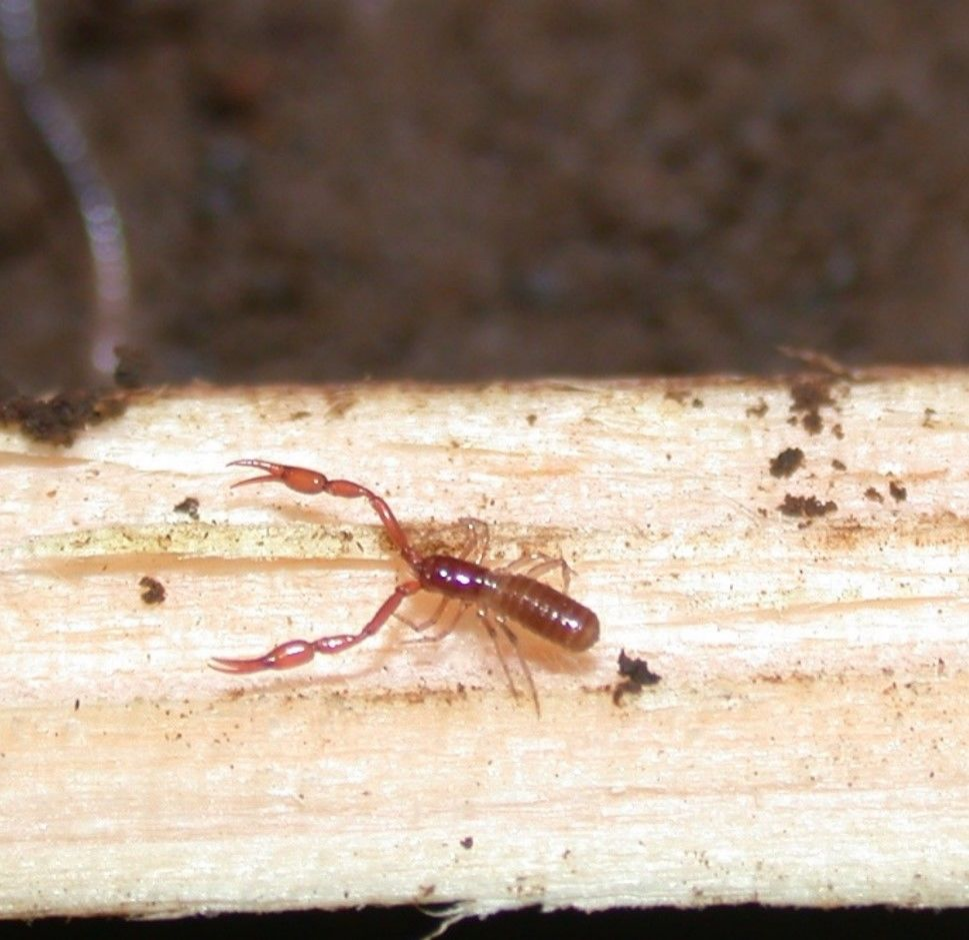
*Pseudoblothrus oromii* (Mahnert, 1990) from S. Jorge (Azores, Portugal) (Credit: Paulo A.V. Borges).

Range description: *Pseudoblothrus oromii* (Fig. 1) is an endemic cave-adapted pseudoscorpion species known from a single island, S. Jorge (Azores, Portugal) (Borges et al. 2010b) and occurs in a single cave, the lava tube of Gruta da Beira.

#### New occurrences

#### Extent of occurrence

EOO (km2): 4

Trend: Decline (inferred)

Justification for trend: No decrease in EOO has been registered, but it is inferred from decline due to the vulnerability of its habitat, associated with the degradation of volcanic caves and anthropogenic impact in the surrounding area. Indeed, the cave Gruta da Beira is suffering from the important impact of dairy cattle management and pollution, and, because the cave is very accessible to visitation, the recreational cave visitation can have some impacts which have still not been measured.

Causes ceased?: No

Causes understood?: Yes

Causes reversible?: Unknown

Extreme fluctuations?: No

#### Area of occupancy

Trend: Decline (inferred)

Justification for trend: No decrease in AOO has been registered, but it is inferred from decline due to the vulnerability of its habitat, associated with the degradation of volcanic caves and anthropogenic impact in the surrounding area (see details above in EOO).

Causes ceased?: No

Causes understood?: Yes

Causes reversible?: Unknown

Extreme fluctuations?: No

AOO (km2): 4

#### Locations

Number of locations: 1

Justification for number of locations: *Pseudoblothrus oromii* occurs in a single cave, the cave of Gruta da Beira on the island of S. Jorge (Azores, Portugal), that is under intense disturbance (see threats below).

Trend: Stable

Justification for trend: The species is historically only known from a single location.

Extreme fluctuations?: No

#### Population

Number of individuals: Unknown

Trend: Decline (inferred)

Justification for trend: The area surrounding the cave is heavily impacted by human disturbance.

Basis for decline: (c) a decline in area of occupancy, extent of occurrence and/or quality of habitat

Causes ceased?: No

Causes understood?: Yes

Causes reversible?: Unknown

Extreme fluctuations?: No

Population Information (Narrative): The species is rare and only known from a single subpopulation in S. Jorge island.

#### Subpopulations

Trend: Decline (observed)

Justification for trend: The area surrounding the cave is heavily impacted by human disturbance.

Extreme fluctuations?: No

Severe fragmentation?: No

#### Habitat

System: Terrestrial

Habitat specialist: Yes

Habitat (narrative): Specimens were found near the entrance of the cave, under rotting wood and other organic litter.

Trend in extent, area or quality?: Decline (observed)

Justification for trend: Gruta da Beira is a 200 m long lava tube opening on a slope near an urbanised area. The surrounding area is highly disturbed by agricultural fields, mostly intensive pasture. The recent increase in tourism in Azores is creating opportunities to more organised visits to this cave that, due to its small size, could be highly impacted by recreational activities.

##### Habitat

Habitat importance: Major Importance

Habitats: 7.1. Caves and Subterranean Habitats (non-aquatic) - Caves

#### Habitat

Habitat importance: Major Importance

Habitats: 7.1. Caves and Subterranean Habitats (non-aquatic) - Caves

#### Ecology

Generation length (yr): 1

Dependency of single sp?: No

Ecology and traits (narrative): The genus *Pseudoblothrus* is exclusively cave-dwelling (Mahnert 1990). *Pseudoblothrus oromii* is a cavernicolous (i.e. a troglobitic species) predator and/or saprophagous species.

#### Threats

Justification for threats: The main current threats to this species are the loss of habitat quality due to the impact of agriculture activities, agricultural and domestic pollution and recreational cave visitation. However, there are several future potential threats: climate changes in many habitats in Azores (see Ferreira et al. 2016) that can change the conditions inside the cave; change in the road infrastructure around the cave; potential human recreational activities with disturbance caused by radical cave visitation.

##### Threats

Threat type: Ongoing

Threats: 1.1. Residential & commercial development - Housing & urban areas2.1.2. Agriculture & aquaculture - Annual & perennial non-timber crops - Small-holder farming2.3. Agriculture & aquaculture - Livestock farming & ranching6.1. Human intrusions & disturbance - Recreational activities9.1.2. Pollution - Domestic & urban waste water - Run-off9.3.1. Pollution - Agricultural & forestry effluents - Nutrient loads9.3.3. Pollution - Agricultural & forestry effluents - Herbicides and pesticides

##### Threats

Threat type: Future

Threats: 4.1. Transportation & service corridors - Roads & railroads11.1. Climate change & severe weather - Habitat shifting & alteration11.2. Climate change & severe weather - Droughts

#### Threats

Threat type: Ongoing

Threats: 1.1. Residential & commercial development - Housing & urban areas2.1.2. Agriculture & aquaculture - Annual & perennial non-timber crops - Small-holder farming2.3. Agriculture & aquaculture - Livestock farming & ranching6.1. Human intrusions & disturbance - Recreational activities9.1.2. Pollution - Domestic & urban waste water - Run-off9.3.1. Pollution - Agricultural & forestry effluents - Nutrient loads9.3.3. Pollution - Agricultural & forestry effluents - Herbicides and pesticides

#### Threats

Threat type: Future

Threats: 4.1. Transportation & service corridors - Roads & railroads11.1. Climate change & severe weather - Habitat shifting & alteration11.2. Climate change & severe weather - Droughts

#### Conservation

Justification for conservation actions: Although the species is protected by regional law (RAA 2012), the cave where it occurs is, however, not protected. Land-use changes are one of the main current and future threats and conservation measures should be extended beyond the cave. As future measure of conservation, the limiting of visits to the cave could be considered. A habitat management plan is needed and anticipated to be developed during the coming years.

##### Conservation actions

Conservation action type: Needed

Conservation actions: 1.1. Land/water protection - Site/area protection2.1. Land/water management - Site/area management2.3. Land/water management - Habitat & natural process restoration5.1.3. Law & policy - Legislation - Sub-national level4.1. Education & awareness - Formal education

#### Conservation actions

Conservation action type: Needed

Conservation actions: 1.1. Land/water protection - Site/area protection2.1. Land/water management - Site/area management2.3. Land/water management - Habitat & natural process restoration5.1.3. Law & policy - Legislation - Sub-national level4.1. Education & awareness - Formal education

#### Other

Justification for use and trade: The species is not utilised.

Justification for ecosystem services : Insufficient information available.

##### Use and trade

Use type: International

##### Ecosystem services

Ecosystem service type: Less important

##### Research needed

Research needed: 1.2. Research - Population size, distribution & trends1.3. Research - Life history & ecology2.2. Conservation Planning - Area-based Management Plan3.1. Monitoring - Population trends

Justification for research needed: Further research is needed into its population, ecology and life history. It is necessary to establish a monitoring plan for the invertebrate community in the habitat in order to contribute to the conservation of this species.

#### Use and trade

Use type: International

#### Ecosystem services

Ecosystem service type: Less important

#### Research needed

Research needed: 1.2. Research - Population size, distribution & trends1.3. Research - Life history & ecology2.2. Conservation Planning - Area-based Management Plan3.1. Monitoring - Population trends

Justification for research needed: Further research is needed into its population, ecology and life history. It is necessary to establish a monitoring plan for the invertebrate community in the habitat in order to contribute to the conservation of this species.

#### Viability analysis

### Pseudoblothrus vulcanus

#### Species information

Scientific name: Pseudoblothrus vulcanus

Species authority: Mahnert, 1990

Kingdom: Animalia

Phylum: Arthropoda

Class: Arachnida

Order: Pseudoscorpion

Family: Syarinidae

Region for assessment: Global

#### Editor & Reviewers

##### Reviewers

Reviewers: Dinarte Teixeira

##### Editor

Editor: Axel Hochkirch

#### Reviewers

Reviewers: Dinarte Teixeira

#### Editor

Editor: Axel Hochkirch

#### Geographic range

Biogeographic realm: Palearctic

Countries: Portugal

Map of records (Google Earth): Suppl. material 4

Basis of EOO and AOO: Known habitat extent

Basis (narrative): The extent of occurrence (EOO) is 1525 km² and the maximum estimated area of occupancy (AOO) is 20 km².

Range description: *Pseudoblothrus vulcanus* is an endemic cave-adapted pseudoscorpion species known from Pico and Terceira islands (Azores, Portugal) (Borges et al. 2010b). Originally described from Gruta das Agulhas (Terceira), it is present in a total of eight caves and lava tubes in both islands; Furna da Baliza, Furna do Frei Matias and Furna Nova (Pico); Gruta das Agulhas, Gruta do Coelho, Gruta da Malha, Gruta dos Principiantes and Gruta de Santa Maria (Terceira).

#### New occurrences

#### Extent of occurrence

EOO (km2): 1525

Trend: Decline (inferred)

Justification for trend: No decrease in EOO has been registered but it is inferred from decline due to the vulnerability of its habitat, associated with the degradation of volcanic caves and anthropogenic impact in the surrounding area. In Furna do Frei Matias, Gruta das Agulhas and Gruta dos Principiantes, there is a current impact due to cave visitation. In almost of all the caves, agricultural activities and livestock raising, agricultural and domestic pollution are major problems for the underground environment.

Causes ceased?: No

Causes understood?: Yes

Causes reversible?: Unknown

Extreme fluctuations?: No

#### Area of occupancy

Trend: Decline (inferred)

Justification for trend: No decrease in AOO has been registered, but it is inferred from decline due to the vulnerability of its habitat, associated with the degradation of volcanic caves and anthropogenic impact in the surrounding area (see details above).

Causes ceased?: No

Causes understood?: Yes

Causes reversible?: Unknown

Extreme fluctuations?: No

AOO (km2): 20

#### Locations

Number of locations: 8

Justification for number of locations: This species occurs in eight volcanic caves in Pico (Furna da Baliza, Furna do Frei Matias and Furna Nova) and Terceira (Gruta das Agulhas, Gruta do Coelho, Gruta da Malha, Gruta dos Principiantes and Gruta de Santa Maria) islands that are under several threats, namely: agricultural activities and livestock raising, agricultural and domestic pollution and recreational cave visitation. Additionally, invasive plant species, altering the habitat at the entrance of the caves, might also impact the overall habitat quality in the caves.

Trend: Decline (inferred)

Justification for trend: Those 8 volcanic caves in Pico and Terceira islands are located in an area heavily impacted by agricultural activities and livestock raising, agricultural and domestic pollution, as well as by recreational cave visitation. Additionally, invasive plant species, altering the habitat at the entrance of the caves, might also impact the overall habitat quality in the caves. Possibly, the species occurred in more caves that have now been destroyed due to agriculture, road and urban development.

Extreme fluctuations?: No

#### Population

Number of individuals: Unknown

Trend: Decline (inferred)

Justification for trend: Due to ongoing impacts on the caves, the impact of human activities decreases the quality of cave environment.

Basis for decline: (c) a decline in area of occupancy, extent of occurrence and/or quality of habitat

Causes ceased?: No

Causes understood?: Yes

Causes reversible?: Unknown

Extreme fluctuations?: No

Population Information (Narrative): This species is apparently quite common, occurring in eight volcanic caves. However, the threats described below are believed to be leading to a decrease in population numbers.

#### Subpopulations

Trend: Decline (inferred)

Justification for trend: The areas surrounding the caves ar heavily impacted by human disturbance.

Extreme fluctuations?: No

Severe fragmentation?: No

#### Habitat

System: Terrestrial

Habitat specialist: Yes

Habitat (narrative): The species occurs in eight lava tubes, some in protected areas (Natural parks of Pico and Terceira), others are surrounded by highly disturbed or urbanised areas and two being coastal caves.

Trend in extent, area or quality?: Decline (observed)

##### Habitat

Habitat importance: Major Importance

Habitats: 7.1. Caves and Subterranean Habitats (non-aquatic) - Caves

#### Habitat

Habitat importance: Major Importance

Habitats: 7.1. Caves and Subterranean Habitats (non-aquatic) - Caves

#### Ecology

Generation length (yr): 1

Dependency of single sp?: No

Ecology and traits (narrative): There is limited information regarding this species ecology and life-history. The genus *Pseudoblothrus* is exclusively cave-dwelling (Mahnert 1990). Specimens were found near the entrance of the caves, under rotting wood and other organic litter. It is a cavernicolous (i.e. a troglobitic species) predator and/or saprophagous species. Its eyes are more developed than those of *P. oromii* (Mahnert 1990).

#### Threats

Justification for threats: The main current threats to this species are the loss of habitat quality due to the impact of agricultural activities and livestock raising, agricultural and domestic pollution and recreational cave visitation. Additionally, invasive plant species, altering the habitat at the entrance of the caves, might also impact the overall habitat quality in the caves. However, there are several future potential threats: climatic changes will impact many habitats in Azores (see Ferreira et al. 2016) and this can change the conditions inside the cave; change in the road infrastructure around the cave; potential human recreational activities with disturbance caused by radical cave visitation.

##### Threats

Threat type: Ongoing

Threats: 1.1. Residential & commercial development - Housing & urban areas2.1.2. Agriculture & aquaculture - Annual & perennial non-timber crops - Small-holder farming2.2.1. Agriculture & aquaculture - Wood & pulp plantations - Small-holder plantations2.3.2. Agriculture & aquaculture - Livestock farming & ranching - Small-holder grazing, ranching or farming6.1. Human intrusions & disturbance - Recreational activities7.3. Natural system modifications - Other ecosystem modifications8.1.1. Invasive and other problematic species, genes & diseases - Invasive non-native/alien species/diseases - Unspecified species9.1.2. Pollution - Domestic & urban waste water - Run-off9.3.1. Pollution - Agricultural & forestry effluents - Nutrient loads9.3.3. Pollution - Agricultural & forestry effluents - Herbicides and pesticides

##### Threats

Threat type: Future

Threats: 4.1. Transportation & service corridors - Roads & railroads11.1. Climate change & severe weather - Habitat shifting & alteration11.2. Climate change & severe weather - Droughts

#### Threats

Threat type: Ongoing

Threats: 1.1. Residential & commercial development - Housing & urban areas2.1.2. Agriculture & aquaculture - Annual & perennial non-timber crops - Small-holder farming2.2.1. Agriculture & aquaculture - Wood & pulp plantations - Small-holder plantations2.3.2. Agriculture & aquaculture - Livestock farming & ranching - Small-holder grazing, ranching or farming6.1. Human intrusions & disturbance - Recreational activities7.3. Natural system modifications - Other ecosystem modifications8.1.1. Invasive and other problematic species, genes & diseases - Invasive non-native/alien species/diseases - Unspecified species9.1.2. Pollution - Domestic & urban waste water - Run-off9.3.1. Pollution - Agricultural & forestry effluents - Nutrient loads9.3.3. Pollution - Agricultural & forestry effluents - Herbicides and pesticides

#### Threats

Threat type: Future

Threats: 4.1. Transportation & service corridors - Roads & railroads11.1. Climate change & severe weather - Habitat shifting & alteration11.2. Climate change & severe weather - Droughts

#### Conservation

Justification for conservation actions: The species is protected by regional law (RAA 2012), as are some of the caves where it occurs (Natural Parks of Pico and Terceira). Land-use changes are one of the main current and future threats and conservation and restoration measures should be extended beyond the caves. As a future measure forconservation, the restriction of visits to the caves could be considered. A habitat management plan is needed and is anticipated to be developed during the coming years. It is necessary to establish a monitoring plan for the invertebrate community in the habitat in order to contribute to the conservation of this species.

##### Conservation actions

Conservation action type: Needed

Conservation actions: 2.1. Land/water management - Site/area management2.2. Land/water management - Invasive/problematic species control2.3. Land/water management - Habitat & natural process restoration3.1. Species management - Species management5. Law & policy4.3. Education & awareness - Awareness & communications

#### Conservation actions

Conservation action type: Needed

Conservation actions: 2.1. Land/water management - Site/area management2.2. Land/water management - Invasive/problematic species control2.3. Land/water management - Habitat & natural process restoration3.1. Species management - Species management5. Law & policy4.3. Education & awareness - Awareness & communications

#### Other

Justification for ecosystem services : Insufficient information available.

##### Use and trade

Use type: International

##### Ecosystem services

Ecosystem service type: Less important

##### Research needed

Research needed: 1.1. Research - Taxonomy2.2. Conservation Planning - Area-based Management Plan3.1. Monitoring - Population trends

Justification for research needed: Further research is needed into its population, ecology and life history. The fact that the species is a cave-adapted species and occurs in two islands may imply that we are in the presence of two cryptic species. Therefore, there is the urgent need of a taxonomic revision of this taxon.

#### Use and trade

Use type: International

#### Ecosystem services

Ecosystem service type: Less important

#### Research needed

Research needed: 1.1. Research - Taxonomy2.2. Conservation Planning - Area-based Management Plan3.1. Monitoring - Population trends

Justification for research needed: Further research is needed into its population, ecology and life history. The fact that the species is a cave-adapted species and occurs in two islands may imply that we are in the presence of two cryptic species. Therefore, there is the urgent need of a taxonomic revision of this taxon.

#### Viability analysis

### Rugathodes pico

#### Species information

Scientific name: Rugathodes pico

Species authority: Merrett & Ashmole, 1989

Kingdom: Animalia

Phylum: Arthropoda

Class: Arachnida

Order: Araneae

Family: Theridiidae

**Figure 2.**
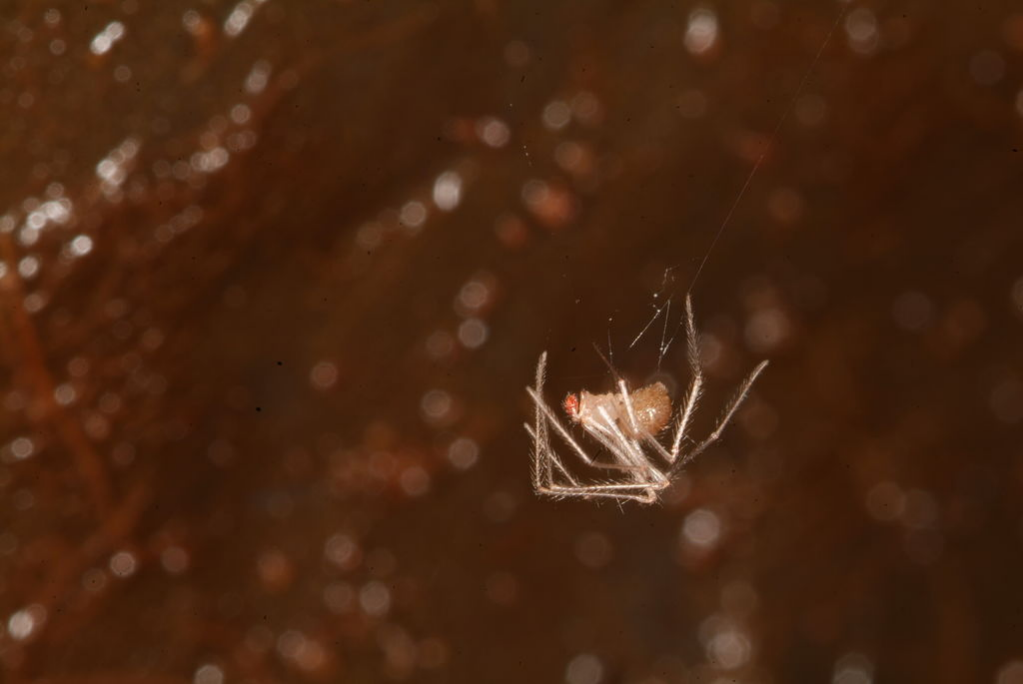
*Rugathodes pico* (Merrett & Ashmole, 1989) from Faial and Pico islands (Azores, Portugal) (Credit: Pedro Cardoso).

Figure(s) or Photo(s): Fig. 2

Region for assessment: Global

#### Editor & Reviewers

##### Reviewers

Reviewers: Sérgio Henriques

##### Editor

Editor: Axel Hochkirch

#### Reviewers

Reviewers: Sérgio Henriques

#### Editor

Editor: Axel Hochkirch

#### Geographic range

Biogeographic realm: Palearctic

Countries: Portugal

Map of records (Google Earth): Suppl. material 5

Basis of EOO and AOO: Observed

Basis (narrative): The extent of occurrence (EOO) is *ca.* 275 km^2^ and the maximum estimated area of occupancy (AOO) is 28 km^2^.

Range description: *Rugathodes pico* is a cave-adapted endemic species known from Pico and Faial (Azores, Portugal) (Borges et al. 2010b), occurring in seven volcanic caves in Faial (Furna Ruim) and Pico (Furna dos Montanheiros, Gruta das Canárias, Gruta da Agostinha, Gruta do Henrique Maciel, Gruta do Mistério da Silveira I, Gruta do Soldão) (Pereira et al. 2015).

#### New occurrences

#### Extent of occurrence

EOO (km2): 275

Trend: Decline (inferred)

Justification for trend: The species is a specialised troglobite living in constant humidity conditions. Many caves in Faial and Pico Islands are being impacted by pollution due to intensive cattle production, with changes in ecological conditions of caves in the last 50 years, namely, the change of the N and P abiotic cycles and changes in the water pH (Hathaway et al. 2014).

Causes ceased?: No

Causes understood?: Yes

Causes reversible?: Unknown

Extreme fluctuations?: No

#### Area of occupancy

Trend: Decline (inferred)

Justification for trend: In Faial and Pico Islands, there are 19 well-surveyed caves and we found subpopulations in only seven. The trend of decline is partly based on the assumption that this species can occur in all these caves and that the absence is due not only to biological reasons (type of cave; age of the lava flow), but mainly to anthropogenic disturbance on caves during the last 50 years. Indeed, there is a strong predominance of intensive pastures and maize in areas theoretically unsuitable for this purpose in many Azorean islands, with the expansion between 2001 and 2011 of pasture intensification to areas more suitable to forest (Reis and Dentinho 2015). Most of the caves were in the past covered by dense humid native forest and forest clearance promoted changes in humidity and resource availability in the cave environment.

Causes ceased?: No

Causes understood?: Yes

Causes reversible?: Unknown

Extreme fluctuations?: No

AOO (km2): 28

#### Locations

Number of locations: 6

Justification for number of locations: Six out of the seven volcanic caves (Gruta da Agostinha, Furna dos Montanheiros, Gruta das Canárias, Gruta do Henrique Maciel, Gruta do Mistério da Silveira I, Gruta do Soldão) (Pereira et al. 2015) are being affected by different threats, mainly touristic pressure, wine and cattle production with consequent deforestation and nutrient input into caves.

Trend: Decline (inferred)

Justification for trend: After a detailed survey of 19 caves in Faial and Pico islands that include most of the range of the species, the species was only found at seven, which is a small number of caves for a predictably larger range (up to 5 times larger) just 50 years ago.

Extreme fluctuations?: No

#### Population

Number of individuals: Unknown

Trend: Decline (inferred)

Justification for trend: Each cave where the species occurs is affected by different threats, mainly touristic pressure, wine and cattle production with consequent deforestation and nutrient input into caves.

Basis for decline: (c) a decline in area of occupancy, extent of occurrence and/or quality of habitat

Causes ceased?: No

Causes understood?: Yes

Causes reversible?: Unknown

Extreme fluctuations?: No

Population Information (Narrative): Seven subpopulations of this species can be found across two islands, but most of them are very small and located in disturbed lava tubes. The single large subpopulation is located in Gruta da Agostinha, which is under future threat due to increasing possibility of land-use changes for wine production.

#### Subpopulations

Number of subpopulations: 7

Trend: Decline (observed)

Justification for trend: Most of the subpopulations are living in volcanic caves surrounded by agricultural activities and/or domestic pollution and we suspect that the species has disappeared from other caves in the region.

Extreme fluctuations?: No

Severe fragmentation?: No

#### Habitat

System: Terrestrial

Habitat specialist: Yes

Habitat (narrative): The species is a troglobite specialist, occurring only in humid lava tubes and volcanic pits. In the cave with the larger subpopulation (Gruta da Agostinha), the species occurs in all sections of the cave.

Trend in extent, area or quality?: Decline (inferred)

Justification for trend: The intensive cattle production in the islands of Faial and Pico has increased considerably in the last twenty years and creates high disturbance and pollution in the cave systems. Touristic pressure and land-use changes to wine production might also be a threat, through reduction in habitat quality.

##### Habitat

Habitat importance: Major Importance

Habitats: 7.1. Caves and Subterranean Habitats (non-aquatic) - Caves

#### Habitat

Habitat importance: Major Importance

Habitats: 7.1. Caves and Subterranean Habitats (non-aquatic) - Caves

#### Ecology

Size: 2.45 mm

Generation length (yr): 1

Dependency of single sp?: No

Ecology and traits (narrative): *T. pico* adaptations related to cave life are the very pale colour, the long spines and hairs and the extreme length of the legs (Merrett and Ashmole 1989). The species builds cobwebs in open spaces and across small holes in the volcanic basaltic rock. Usually occurs from twilight conditions near cave openings to deep parts of the caves.

#### Threats

Justification for threats: The main current threat to this species is the impact of agricultural activities, namely the expansion of wine production and domestic pollution. However, there are several future potential threats: climatic changes will impact many habitats in the Azores (see Ferreira et al. 2016) and this can change the conditions inside the caves; urban development in coastal areas, changes in the road infrastructure around the caves; logging of *Pittosporum undulatum* exotic forests over the caves; potential human recreational activities with disturbance caused by radical cave visitation.

##### Threats

Threat type: Ongoing

Threats: 2.1.3. Agriculture & aquaculture - Annual & perennial non-timber crops - Agro-industry farming2.3.2. Agriculture & aquaculture - Livestock farming & ranching - Small-holder grazing, ranching or farming6.1. Human intrusions & disturbance - Recreational activities9.1.1. Pollution - Domestic & urban waste water - Sewage

##### Threats

Threat type: Future

Threats: 1.1. Residential & commercial development - Housing & urban areas4.1. Transportation & service corridors - Roads & railroads11.1. Climate change & severe weather - Habitat shifting & alteration11.2. Climate change & severe weather - Droughts5.3.2. Biological resource use - Logging & wood harvesting - Intentional use (large scale)

#### Threats

Threat type: Ongoing

Threats: 2.1.3. Agriculture & aquaculture - Annual & perennial non-timber crops - Agro-industry farming2.3.2. Agriculture & aquaculture - Livestock farming & ranching - Small-holder grazing, ranching or farming6.1. Human intrusions & disturbance - Recreational activities9.1.1. Pollution - Domestic & urban waste water - Sewage

#### Threats

Threat type: Future

Threats: 1.1. Residential & commercial development - Housing & urban areas4.1. Transportation & service corridors - Roads & railroads11.1. Climate change & severe weather - Habitat shifting & alteration11.2. Climate change & severe weather - Droughts5.3.2. Biological resource use - Logging & wood harvesting - Intentional use (large scale)

#### Conservation

Justification for conservation actions: The species is not protected by regional law. Some of the caves are included in the Natural Park of Faial and Pico. Since land-use changes (for *Pittosporum undulatum* removal, urban development, wine production) is the main current and future threat, it might be important to safeguard the species survival in the future and conservation should be extended beyond the current area, possibly allowing the recovery of other caves to original conditions where the species might be reintroduced. The addition of fences around the caves will be an important mitigation measure.

##### Conservation actions

Conservation action type: Needed

Conservation actions: 1.2. Land/water protection - Resource & habitat protection2.1. Land/water management - Site/area management3.3.1. Species management - Species re-introduction - Reintroduction5.1.3. Law & policy - Legislation - Sub-national level4.1. Education & awareness - Formal education

#### Conservation actions

Conservation action type: Needed

Conservation actions: 1.2. Land/water protection - Resource & habitat protection2.1. Land/water management - Site/area management3.3.1. Species management - Species re-introduction - Reintroduction5.1.3. Law & policy - Legislation - Sub-national level4.1. Education & awareness - Formal education

#### Other

Justification for use and trade: The species is not utilised.

##### Use and trade

Use type: International

##### Ecosystem services

Ecosystem service type: Very important

Ecosystem services: 12. Biocontrol

##### Research needed

Research needed: 1.2. Research - Population size, distribution & trends1.3. Research - Life history & ecology2.1. Conservation Planning - Species Action/Recovery Plan2.2. Conservation Planning - Area-based Management Plan3.1. Monitoring - Population trends3.4. Monitoring - Habitat trends

Justification for research needed: Further research is needed into its ecology and life history in order to find extant specimens in additional caves. An area-based management plan is necessary for the most disturbed caves including invertebrate monitoring to contribute to a potential species recovery plan.

#### Use and trade

Use type: International

#### Ecosystem services

Ecosystem service type: Very important

Ecosystem services: 12. Biocontrol

#### Research needed

Research needed: 1.2. Research - Population size, distribution & trends1.3. Research - Life history & ecology2.1. Conservation Planning - Species Action/Recovery Plan2.2. Conservation Planning - Area-based Management Plan3.1. Monitoring - Population trends3.4. Monitoring - Habitat trends

Justification for research needed: Further research is needed into its ecology and life history in order to find extant specimens in additional caves. An area-based management plan is necessary for the most disturbed caves including invertebrate monitoring to contribute to a potential species recovery plan.

#### Viability analysis

### Macarorchestia martini

#### Species information

Scientific name: Macarorchestia martini

Species authority: Stock, 1989

Kingdom: Animalia

Phylum: Arthropoda

Class: Malacostraca

Order: Amphipoda

Family: Talitridae

Region for assessment: Global

#### Editor & Reviewers

##### Reviewers

Reviewers: Dinarte Teixeira

##### Editor

Editor: Axel Hochkirch

#### Reviewers

Reviewers: Dinarte Teixeira

#### Editor

Editor: Axel Hochkirch

#### Geographic range

Biogeographic realm: Palearctic

Countries: Portugal

Map of records (Google Earth): Suppl. material 1

Basis of EOO and AOO: Known habitat extent

Basis (narrative): The extent of occurrence (EOO) is 4 km² and the maximum estimated area of occupancy (AOO) is 4 km².

Min Elevation/Depth (m): 5

Max Elevation/Depth (m): 5

Range description: *Macarorchestia martini* is possibly an endemic cave-adapted species known from a single island, Terceira (Azores, Portugal) (Borges et al. 2010b) and occurs in a single cave, the coastal lava tube of Gruta das Agulhas. Additional surveys are needed to confirm the troglobiont status of this species (see e.g. Wildish 2014).

#### New occurrences

#### Extent of occurrence

EOO (km2): 4

Trend: Decline (inferred)

Justification for trend: The species is occurring in a single cave, the coastal lava tube of Gruta das Agulhas, Terceira (Azores, Portugal). No decrease in EOO has been registered but it is inferred from decline due to the vulnerability of its habitat, associated with the degradation of volcanic caves and anthropogenic impact in the surrounding area (e.g. agriculture pollution).

Causes ceased?: No

Causes understood?: Yes

Causes reversible?: Unknown

Extreme fluctuations?: No

#### Area of occupancy

Trend: Decline (inferred)

Justification for trend: This species occurs only in a cave of Terceira island (Gruta das Agulhas) and it is not known if it ever occurred outside it. No decrease in AOO was observed, but it is inferred from decline in habitat quality associated with the degradation of volcanic caves and anthropogenic impact in the surrounding area (e.g. agriculture pollution).

Causes ceased?: No

Causes understood?: Yes

Causes reversible?: Unknown

Extreme fluctuations?: No

AOO (km2): 4

#### Locations

Number of locations: 1

Justification for number of locations: The species is only known on a single island, Terceira (Azores, Portugal) (Borges et al. 2010b) and occurs in a single cave, the coastal lava tube of Gruta das Agulhas, that is heavily impacted by human disturbance. The aboveground area is disturbed by urbanisation and agricultural fields.

Trend: Stable

Justification for trend: The species is only present in one single cave. Stable despite the impending threats.

Extreme fluctuations?: No

#### Population

Number of individuals: Unknown

Trend: Decline (inferred)

Justification for trend: The area surrounding the cave is heavily impacted by human disturbance and decreases the quality of the cave environment.

Basis for decline: (c) a decline in area of occupancy, extent of occurrence and/or quality of habitat

Causes ceased?: No

Causes understood?: Yes

Causes reversible?: Unknown

Extreme fluctuations?: Unknown

Population Information (Narrative): The species is rare and only known from a single subpopulation in Terceira island. The area surrounding the cave is heavily impacted by human disturbance.

#### Subpopulations

Trend: Stable

Justification for trend: Only one subpopulation historically known.

Extreme fluctuations?: No

Justification for extreme fluctuations: The species occurs naturally in a single cave.

Severe fragmentation?: No

#### Habitat

System: Terrestrial

Habitat specialist: Yes

Habitat (narrative): Specimens were found at some distance from the entrance of Gruta das Agulhas (a 250 m long lava tube on the seashore, opening some 5 m above the sea level), but where dim light was still available, in high humidity but without permanent water.

Trend in extent, area or quality?: Decline (inferred)

Justification for trend: The area surrounding the cave is heavily impacted by human disturbance; the aboveground area is disturbed by urbanisation and agricultural fields (pollution by herbicides and pesticides). The easy access to the cave leads to uncontrolled cave visitation with potential impacts on the quality of the ecosystem. Debris is frequently seen in the cave.

##### Habitat

Habitat importance: Major Importance

Habitats: 7.1. Caves and Subterranean Habitats (non-aquatic) - Caves13.2. Marine Coastal/Supratidal - Coastal Caves/Karst

#### Habitat

Habitat importance: Major Importance

Habitats: 7.1. Caves and Subterranean Habitats (non-aquatic) - Caves13.2. Marine Coastal/Supratidal - Coastal Caves/Karst

#### Ecology

Size: 5-6 mm

Generation length (yr): 1

Dependency of single sp?: No

Ecology and traits (narrative): There is limited information regarding this species ecology and life history. This species has reduced eyes, but few other adaptations to cave life (Stock 1989). Mentioned as trogloxene, even as a driftwood specialist in Wildish 2014 .

#### Threats

Justification for threats: The main current threats to this species are the loss of habitat quality at the cave entrance due to the impact of agriculture activities, agricultural and domestic pollution and recreational cave visitation. However, there are several future potential threats: climatic changes (see Ferreira et al. 2016) are expected to cause habitat changes in lower elevations in the Azores, that can change the conditions inside the cave, particularly at the cave entrance where the species occurs; change in the road infrastructure around the cave; expanding urban development in the coastal area. The current increase in tourism in the Azores is promoting the increase in cave visitation and uncontrolled recreational activities with expected disturbance caused by radical cave visitation.

##### Threats

Threat type: Ongoing

Threats: 1.1. Residential & commercial development - Housing & urban areas6.1. Human intrusions & disturbance - Recreational activities9.1.1. Pollution - Domestic & urban waste water - Sewage9.1.3. Pollution - Domestic & urban waste water - Type Unknown/Unrecorded9.3.3. Pollution - Agricultural & forestry effluents - Herbicides and pesticides

##### Threats

Threat type: Future

Threats: 4.1. Transportation & service corridors - Roads & railroads6.1. Human intrusions & disturbance - Recreational activities11.1. Climate change & severe weather - Habitat shifting & alteration11.2. Climate change & severe weather - Droughts

#### Threats

Threat type: Ongoing

Threats: 1.1. Residential & commercial development - Housing & urban areas6.1. Human intrusions & disturbance - Recreational activities9.1.1. Pollution - Domestic & urban waste water - Sewage9.1.3. Pollution - Domestic & urban waste water - Type Unknown/Unrecorded9.3.3. Pollution - Agricultural & forestry effluents - Herbicides and pesticides

#### Threats

Threat type: Future

Threats: 4.1. Transportation & service corridors - Roads & railroads6.1. Human intrusions & disturbance - Recreational activities11.1. Climate change & severe weather - Habitat shifting & alteration11.2. Climate change & severe weather - Droughts

#### Conservation

Justification for conservation actions: Although the species is protected by regional law (RAA 2012), the cave where it occurs is, however, not protected. Land-use changes are one of the main current and future threats and conservation measures should be extended beyond the cave. As a future measure of conservation, the restriction of visits to the cave could be considered. A habitat management plan is needed and is anticipated to be developed during the coming years.

##### Conservation actions

Conservation action type: Needed

Conservation actions: 2.1. Land/water management - Site/area management2.3. Land/water management - Habitat & natural process restoration3.1. Species management - Species management4. Education & awareness

#### Conservation actions

Conservation action type: Needed

Conservation actions: 2.1. Land/water management - Site/area management2.3. Land/water management - Habitat & natural process restoration3.1. Species management - Species management4. Education & awareness

#### Other

Justification for use and trade: The species is not utilised.

Justification for ecosystem services : Insufficient information available.

##### Use and trade

Use type: International

##### Ecosystem services

Ecosystem service type: Less important

##### Research needed

Research needed: 1.2. Research - Population size, distribution & trends1.3. Research - Life history & ecology2.2. Conservation Planning - Area-based Management Plan3.1. Monitoring - Population trends3.4. Monitoring - Habitat trends

Justification for research needed: Further research is needed into its ecology and life history in order to provide a species conservation plan and a management plan that would improve the survival chances of this species for the future. A species conservation plan and a management plan would improve its survival chances for the future. Additional surveys are needed to confirm the troglobiont status of this species (see e.g. Wildish 2014).

#### Use and trade

Use type: International

#### Ecosystem services

Ecosystem service type: Less important

#### Research needed

Research needed: 1.2. Research - Population size, distribution & trends1.3. Research - Life history & ecology2.2. Conservation Planning - Area-based Management Plan3.1. Monitoring - Population trends3.4. Monitoring - Habitat trends

Justification for research needed: Further research is needed into its ecology and life history in order to provide a species conservation plan and a management plan that would improve the survival chances of this species for the future. A species conservation plan and a management plan would improve its survival chances for the future. Additional surveys are needed to confirm the troglobiont status of this species (see e.g. Wildish 2014).

#### Viability analysis

### Pseudosinella ashmoleorum

#### Species information

Scientific name: Pseudosinella ashmoleorum

Species authority: Gama, 1988

Common names: Cave dwelling springtail

Kingdom: Animalia

Phylum: Arthropoda

Class: Hexapoda

Order: Collembola

Family: Entomobryidae

Region for assessment: Global

#### Editor & Reviewers

##### Reviewers

Reviewers: Dinarte Teixeira

##### Editor

Editor: Axel Hochkirch

#### Reviewers

Reviewers: Dinarte Teixeira

#### Editor

Editor: Axel Hochkirch

#### Geographic range

Biogeographic realm: Palearctic

Countries: Portugal

Map of records (Google Earth): Suppl. material 2

Basis of EOO and AOO: Observed

Basis (narrative): The extent of occurrence (EOO) is ca. 2828 km² and the maximum estimated area of occupancy (AOO) is 52 km².

Range description: *Pseudosinella ashmoleorum* is an endemic cave-dwelling springtail species known from Faial, Pico and Terceira islands (Azores, Portugal) (Borges et al. 2010b), known from several caves and lava tubes in Faial (Furna Ruim); in Pico (Gruta da Agostinha, Gruta do Henrique Maciel, Gruta do Soldão); and in Terceira (Algar do Carvão, Gruta das Agulhas, Gruta dos Balcões, Gruta da Caldeira, Gruta do Coelho, Gruta do Chocolate, Gruta da Madre de Deus). The species was also found in the MSS (”Milieu Souterrain Superficiel“ or ”Mesocavernous Shallow Stratum“) (Borges 1993) in the area of Pico Rachado (Terceira island), far from its known distribution in lava caves.

#### New occurrences

#### Extent of occurrence

EOO (km2): 2828

Trend: Stable

Justification for trend: The species occurs naturally in many caves and also in the MSS.

Causes ceased?: No

Causes understood?: Yes

Causes reversible?: Unknown

Extreme fluctuations?: Unknown

#### Area of occupancy

Trend: Stable

Justification for trend: The species occurs naturally in many caves and also in the MSS.

Causes ceased?: No

Causes understood?: Yes

Causes reversible?: Unknown

Extreme fluctuations?: Unknown

AOO (km2): 52

#### Locations

Number of locations: 11

Justification for number of locations: The species occurs in several caves and lava tubes in Faial (Furna Ruim); in Pico (Gruta da Agostinha, Gruta do Henrique Maciel, Gruta do Soldão); in Terceira (Algar do Carvão, Gruta das Agulhas, Gruta dos Balcões, Gruta do Caldeira, Gruta do Coelho, Gruta do Chocolate, Gruta da Madre de Deus) that are under the impact of several important threats, namely: climatic changes in several important habitats in Azores (see Ferreira et al. 2016) that can change the conditions inside the caves; change in the road and urban infrastructure around the caves; potential human recreational activities with disturbance caused by radical cave visitation; reforestation of the areas with exotic trees with unknown impact.

Trend: Stable

Justification for trend: The species is known to be present in 11 caves in 3 different islands that are under the impact of several important threats. The possible additional locations were lost more than 10 years ago, meaning the current trend in number of locations is probably stable despite the impending threats.

Extreme fluctuations?: Unknown

#### Population

Number of individuals: Unknown

Trend: Decline (inferred)

Justification for trend: The decline in number of individuals is inferred from the decline of habitat quality in many caves. Indeed, the high level of agriculture pollution is dramatically changing the cave ecosystems.

Basis for decline: (c) a decline in area of occupancy, extent of occurrence and/or quality of habitat

Causes ceased?: No

Causes understood?: Yes

Causes reversible?: Unknown

Extreme fluctuations?: No

Population Information (Narrative): No current population size estimates exist for this species, but it seems to be relatively widespread through several caves and in the MSS of three islands. The areas surrounding the caves are heavily impacted by human disturbance, including nitrates pollution.

#### Subpopulations

Trend: Decline (inferred)

Justification for trend: The decline in number of subpopulations is inferred from the decline of habitat quality in many caves.

Extreme fluctuations?: No

Severe fragmentation?: No

#### Habitat

System: Terrestrial

Habitat specialist: No

Habitat (narrative): There is limited information regarding this species ecology and life history. It occurs in eleven volcanic caves, some in protected areas (Natural Parks of Pico and Terceira) and some surrounded by disturbed habitats and also in the MSS (mesocavernous shallow stratum) habitats (Borges 1993). Nevertheless, this species has only small possible adaptations to a troglobiont life-style, being likely an eutroglophile (i.e. epigean species able to maintain a permanent subterranean population).

Trend in extent, area or quality?: Decline (inferred)

Justification for trend: The quality of the habitat in most of the caves is decreasing due to the impact of several important threats, namely: agriculture pollution; change in the road and urban infrastructure around the caves; potential human recreational activities with disturbance caused by radical cave visitation; reforestation of the areas with exotic trees with unknown impact.

##### Habitat

Habitat importance: Suitable

Habitats: 7.1. Caves and Subterranean Habitats (non-aquatic) - Caves7.2. Caves and Subterranean Habitats (non-aquatic) - Other Subterranean Habitats

#### Habitat

Habitat importance: Suitable

Habitats: 7.1. Caves and Subterranean Habitats (non-aquatic) - Caves7.2. Caves and Subterranean Habitats (non-aquatic) - Other Subterranean Habitats

#### Ecology

Size: 1.7-2.2 mm

Generation length (yr): 1

Dependency of single sp?: No

Ecology and traits (narrative): *Pseudosinella ashmoleorum* was found in the dark and humid part of caves, with abundant mud or roots from the ceiling (Gama 1988), suggesting the species to be saprophagous.

#### Threats

Justification for threats: The main current threats to this species are the loss of habitat quality due to human activities like agriculture pollution, urbanisation and construction and recreational cave visitation. However, there are several future potential threats: climatic changes will impact many habitats in the Azores (see Ferreira et al. 2016) and this can change the conditions inside the caves, but also changes in the nearby infrastructure, changes in land use, potential human recreational activities with disturbance caused by radical cave visitation.

##### Threats

Threat type: Ongoing

Threats: 1.1. Residential & commercial development - Housing & urban areas2.1.2. Agriculture & aquaculture - Annual & perennial non-timber crops - Small-holder farming2.2.1. Agriculture & aquaculture - Wood & pulp plantations - Small-holder plantations2.3.2. Agriculture & aquaculture - Livestock farming & ranching - Small-holder grazing, ranching or farming6.1. Human intrusions & disturbance - Recreational activities9.3.3. Pollution - Agricultural & forestry effluents - Herbicides and pesticides

##### Threats

Threat type: Future

Threats: 4.1. Transportation & service corridors - Roads & railroads11.1. Climate change & severe weather - Habitat shifting & alteration11.2. Climate change & severe weather - Droughts

#### Threats

Threat type: Ongoing

Threats: 1.1. Residential & commercial development - Housing & urban areas2.1.2. Agriculture & aquaculture - Annual & perennial non-timber crops - Small-holder farming2.2.1. Agriculture & aquaculture - Wood & pulp plantations - Small-holder plantations2.3.2. Agriculture & aquaculture - Livestock farming & ranching - Small-holder grazing, ranching or farming6.1. Human intrusions & disturbance - Recreational activities9.3.3. Pollution - Agricultural & forestry effluents - Herbicides and pesticides

#### Threats

Threat type: Future

Threats: 4.1. Transportation & service corridors - Roads & railroads11.1. Climate change & severe weather - Habitat shifting & alteration11.2. Climate change & severe weather - Droughts

#### Conservation

Justification for conservation actions: The species is not protected by regional law. Part of its habitat is in regionally protected areas (Natural Parks of Pico and Terceira). Land-use change is one of the main current and future threats and conservation and restoration measures should be extended beyond the caves. As a future measure of conservation, the restriction of visits to the caves could be considered. A habitat management plan is needed and is anticipated to be developed during the coming years.

##### Conservation actions

Conservation action type: Needed

Conservation actions: 2.1. Land/water management - Site/area management5.4.3. Law & policy - Compliance and enforcement - Sub-national level4.3. Education & awareness - Awareness & communications

#### Conservation actions

Conservation action type: Needed

Conservation actions: 2.1. Land/water management - Site/area management5.4.3. Law & policy - Compliance and enforcement - Sub-national level4.3. Education & awareness - Awareness & communications

#### Other

Justification for use and trade: The species is not utilised.

Justification for ecosystem services : Insufficient information available.

##### Use and trade

Use type: International

##### Ecosystem services

Ecosystem service type: Less important

##### Research needed

Research needed: 1.2. Research - Population size, distribution & trends1.3. Research - Life history & ecology1.5. Research - Threats2.2. Conservation Planning - Area-based Management Plan3.1. Monitoring - Population trends3.4. Monitoring - Habitat trends

Justification for research needed: Additional research is needed in order to determine the levels of population size, as well as its ecology and life history. It is also necessary to establish a monitoring plan for the invertebrate community in the habitat in order to contribute to the conservation of this species.

#### Use and trade

Use type: International

#### Ecosystem services

Ecosystem service type: Less important

#### Research needed

Research needed: 1.2. Research - Population size, distribution & trends1.3. Research - Life history & ecology1.5. Research - Threats2.2. Conservation Planning - Area-based Management Plan3.1. Monitoring - Population trends3.4. Monitoring - Habitat trends

Justification for research needed: Additional research is needed in order to determine the levels of population size, as well as its ecology and life history. It is also necessary to establish a monitoring plan for the invertebrate community in the habitat in order to contribute to the conservation of this species.

#### Viability analysis

### Cixius cavazoricus

#### Species information

Scientific name: Cixius cavazoricus

Species authority: Hoch, 1991

Common names: Cave planthopper

Kingdom: Animalia

Phylum: Arthropoda

Class: Insecta

Order: Hemiptera Fulgoromorpha

Family: Cixiidae

Figure(s) or Photo(s): 

Region for assessment: Global

#### Editor & Reviewers

##### Reviewers

Reviewers: Anja Danielczak

##### Editor

Editor: Axel Hochkirch

#### Reviewers

Reviewers: Anja Danielczak

#### Editor

Editor: Axel Hochkirch

#### Geographic range

Biogeographic realm: Palearctic

Countries: Portugal

Map of records (Google Earth): Suppl. material 6

Basis of EOO and AOO: Observed

Basis (narrative): The extent of occurrence (EOO) is 8 km^2^ and the maximum estimated area of occupancy (AOO) is 8 km^2^

Min Elevation/Depth (m): 150

Max Elevation/Depth (m): 350

Range description: *Cixius cavazoricus* is a single island endemic cave planthopper species restricted to Faial Island (Azores, Portugal) (Borges et al. 2010b). The species only occurs in two small caves (Gruta das Anelares and Gruta do Cabeço do Canto) (Pereira et al. 2015) and recent fieldwork failed to find it in one of the caves.

#### New occurrences

#### Extent of occurrence

EOO (km2): 8

Trend: Decline (inferred)

Justification for trend: No decrease in EOO has been registered but it is inferred from decline in the habitat quality.

Causes ceased?: No

Causes understood?: Yes

Causes reversible?: Unknown

Extreme fluctuations?: No

#### Area of occupancy

Trend: Decline (inferred)

Justification for trend: No decrease in AOO has been registered but it is inferred from decline in the habitat quality.

Causes ceased?: No

Causes understood?: Yes

Causes reversible?: Unknown

Extreme fluctuations?: No

AOO (km2): 8

#### Locations

Number of locations: 2

Justification for number of locations: Two locations, Gruta das Anelares and Gruta do Cabeço do Canto, are threatened by above-cave deforestation which will reduce the amount of roots suitable as food resources. Increasing nitrogen levels derived from the use of pesticides in neighbouring agricultural land are also a threat since they change the necessary cover by native trees and shrubs above ground.

Trend: Stable

Justification for trend: The current trend in number of locations is probably stable despite the impending threats.

Extreme fluctuations?: No

#### Population

Number of individuals: Unknown

Trend: Decline (estimated)

Justification for trend: The current threats are believed to cause a decrease in the species population numbers at unknown rates.

Basis for decline: (c) a decline in area of occupancy, extent of occurrence and/or quality of habitat

Causes ceased?: No

Causes understood?: Yes

Causes reversible?: Unknown

Extreme fluctuations?: No

Population Information (Narrative): *Cixius cavazoricus* is an obligate cave species known to occur in two lava tubes in Faial Island (Azores, Portugal). The species is extremely rare since only a few specimens have been found despite the sampling efforts. The species was not found during a fieldwork survey in 2010 and only a single specimen was found in one of the caves in May 2014.

#### Subpopulations

Trend: Decline (estimated)

Justification for trend: Only two subpopulations historically known. The species was not found during a fieldwork survey in 2010 and only a single specimen was found in one of the caves in May 2014.

Extreme fluctuations?: No

Severe fragmentation?: No

#### Habitat

System: Terrestrial

Habitat specialist: Yes

Habitat (narrative): This is a troglobitic species that only occurs in two lava tubes in Faial Island (Azores, Portugal) (Hoch 1991).

Trend in extent, area or quality?: Decline (observed)

Justification for trend: Habitat quality is decreasing due to above-cave deforestation which reduces the food resources. Increasing nitrogen levels derived from the use of pesticides in neighbouring agricultural land are also a threat since they change the necessary cover by native trees and shrubs above ground.

##### Habitat

Habitat importance: Major Importance

Habitats: 7.1. Caves and Subterranean Habitats (non-aquatic) - Caves

#### Habitat

Habitat importance: Major Importance

Habitats: 7.1. Caves and Subterranean Habitats (non-aquatic) - Caves

#### Ecology

Size: 4 mm

Generation length (yr): 1

Dependency of single sp?: No

Ecology and traits (narrative): The species is restricted to the deep dark cave zone (Hoch 1991), presents low dispersal ability and it is known to feed on roots of the above-cave vegetation.

#### Threats

Justification for threats: *Cixius cavazoricus* is highly threatened by above-cave deforestation which reduces the food resources. Increasing pesticides in neighbouring agricultural land are also a threat. However, there are also several future potential threats: climatic changes will impact many habitats in the Azores (see Ferreira et al. 2016) and this can change the conditions inside the caves; change in the road infrastructure around the caves; potential human recreational activities with disturbance caused by radical cave visitation.

##### Threats

Threat type: Ongoing

Threats: 2.3.2. Agriculture & aquaculture - Livestock farming & ranching - Small-holder grazing, ranching or farming2.3.3. Agriculture & aquaculture - Livestock farming & ranching - Agro-industry grazing, ranching or farming9.3.3. Pollution - Agricultural & forestry effluents - Herbicides and pesticides

##### Threats

Threat type: Future

Threats: 4.1. Transportation & service corridors - Roads & railroads6.1. Human intrusions & disturbance - Recreational activities11.2. Climate change & severe weather - Droughts11.3. Climate change & severe weather - Temperature extremes

#### Threats

Threat type: Ongoing

Threats: 2.3.2. Agriculture & aquaculture - Livestock farming & ranching - Small-holder grazing, ranching or farming2.3.3. Agriculture & aquaculture - Livestock farming & ranching - Agro-industry grazing, ranching or farming9.3.3. Pollution - Agricultural & forestry effluents - Herbicides and pesticides

#### Threats

Threat type: Future

Threats: 4.1. Transportation & service corridors - Roads & railroads6.1. Human intrusions & disturbance - Recreational activities11.2. Climate change & severe weather - Droughts11.3. Climate change & severe weather - Temperature extremes

#### Conservation

Justification for conservation actions: The species is protected by regional law (RAA 2012) and one of the caves is in the Natural Park of Faial. Monitoring is needed to assess population trends and confirm if the species is no longer present in one of its locations. Measures should be taken to prevent deforestation in the areas above the caves where this species occurs to prevent reduction of food resources for this species. A habitat management plan is needed and is anticipated to be developed during the coming years.

##### Conservation actions

Conservation action type: Needed

Conservation actions: 2.1. Land/water management - Site/area management2.3. Land/water management - Habitat & natural process restoration5.4.3. Law & policy - Compliance and enforcement - Sub-national level4.1. Education & awareness - Formal education4.3. Education & awareness - Awareness & communications

#### Conservation actions

Conservation action type: Needed

Conservation actions: 2.1. Land/water management - Site/area management2.3. Land/water management - Habitat & natural process restoration5.4.3. Law & policy - Compliance and enforcement - Sub-national level4.1. Education & awareness - Formal education4.3. Education & awareness - Awareness & communications

#### Other

Justification for ecosystem services : Insufficient information available.

##### Use and trade

Use type: International

##### Ecosystem services

Ecosystem service type: Less important

##### Research needed

Research needed: 1.2. Research - Population size, distribution & trends1.3. Research - Life history & ecology2.2. Conservation Planning - Area-based Management Plan3.1. Monitoring - Population trends3.4. Monitoring - Habitat trends

Justification for research needed: Further research is needed into its ecology and life history in order to find extant specimens in additional caves. A species conservation plan and a management plan would improve the survival chances of this species for the future.

#### Use and trade

Use type: International

#### Ecosystem services

Ecosystem service type: Less important

#### Research needed

Research needed: 1.2. Research - Population size, distribution & trends1.3. Research - Life history & ecology2.2. Conservation Planning - Area-based Management Plan3.1. Monitoring - Population trends3.4. Monitoring - Habitat trends

Justification for research needed: Further research is needed into its ecology and life history in order to find extant specimens in additional caves. A species conservation plan and a management plan would improve the survival chances of this species for the future.

#### Viability analysis

### Cixius azopicavus

#### Species information

Scientific name: Cixius azopicavus

Species authority: Hoch, 1991

Common names: Cave planthopper

Kingdom: Animalia

Phylum: Arthropoda

Class: Insecta

Order: Hemiptera Fulgoromorpha

Family: Cixiidae

**Figure 3.**
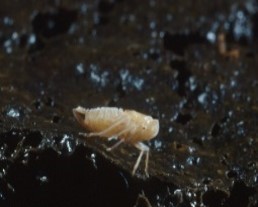
*Cixius azopicavus* (Hoch, 1991) from Pico island (Azores, Portugal) (Credit: Pedro Oromí).

Figure(s) or Photo(s): Fig. 3

Region for assessment: Global

#### Editor & Reviewers

##### Reviewers

Reviewers: Anja Danielczak

##### Editor

Editor: Axel Hochkirch

#### Reviewers

Reviewers: Anja Danielczak

#### Editor

Editor: Axel Hochkirch

#### Geographic range

Biogeographic realm: Palearctic

Countries: Portugal

Map of records (Google Earth): Suppl. material 7

Basis of EOO and AOO: Observed

Basis (narrative): The extent of occurrence (EOO) is 140 km^2^ and the maximum estimated area of occupancy (AOO) is 40 km^2^

Min Elevation/Depth (m): 10

Max Elevation/Depth (m): 770

Range description: *Cixius azopicavus* is a single island endemic cave planthopper species from Pico Island (Azores, Portugal) (Borges et al. 2010b), where it is restricted to six lava tubes (Furna dos Montanheiros, Gruta das Canárias, Gruta da Agostinha, Gruta do Mistério da Silveira I, Gruta do Soldão and Gruta das Torres).

#### New occurrences

#### Extent of occurrence

EOO (km2): 140

Trend: Decline (inferred)

Justification for trend: No decrease in EOO has been registered but it is inferred from decline in the habitat quality.

Causes ceased?: No

Causes understood?: Yes

Causes reversible?: Unknown

Extreme fluctuations?: No

#### Area of occupancy

Trend: Decline (inferred)

Justification for trend: No decrease in AOO has been registered but it is inferred from decline in the habitat quality, including deforestation above the lava tubes, decreasing the amount of roots available, increased nitrogen concentration from the use of pesticides in nearby pastures and increased dryness inside the caves.

Causes ceased?: No

Causes understood?: Yes

Causes reversible?: Unknown

Extreme fluctuations?: No

AOO (km2): 40

#### Locations

Number of locations: 6

Justification for number of locations: Six locations, Furna dos Montanheiros, Gruta das Canárias, Gruta da Agostinha, Gruta do Mistério da Silveira I, Gruta do Soldão and Gruta das Torres, are threatened by above-cave deforestation which will reduce the amount of roots suitable as food resources. Increasing pesticide levels, derived from neighbouring agricultural land, are also a threat.

Trend: Stable

Justification for trend: The current trend in number of locations is probably stable despite the impending threats.

Extreme fluctuations?: No

#### Population

Number of individuals: Unknown

Trend: Decline (inferred)

Justification for trend: The current threats are believed to cause a decrease in the species population numbers at unknown rates.

Basis for decline: (c) a decline in area of occupancy, extent of occurrence and/or quality of habitat

Causes ceased?: No

Causes understood?: Yes

Causes reversible?: Unknown

Extreme fluctuations?: No

Population Information (Narrative): The species only occurs in six lava tubes (caves) in Pico Island (Azores) and is particularly rare in terms of abundance in all caves.

#### Subpopulations

Trend: Decline (inferred)

Justification for trend: Only six subpopulations historically known and some are in danger of extinction. The recent trend in removing exotic and native trees and shrubs for wine fields can be a major threat.

Extreme fluctuations?: No

Severe fragmentation?: No

#### Habitat

System: Terrestrial

Habitat specialist: Yes

Habitat (narrative): This is a troglobitic species with low dispersal ability (Hoch 1991), that occurs in lava tubes with aboveground cover by trees and shrubs. In Furna dos Montanheiros and Gruta das Torres that are located at higher elevations, the dominant vegetation is native, however in the caves located at lower elevations, the vegetation is dominated by the invasive tree *Pittosporum undulatum*.

Trend in extent, area or quality?: Decline (observed)

Justification for trend: Threats to this species include deforestation above the lava tubes, decreasing the amount of roots available, increased pesticides in nearby pastures and increased dryness inside the caves.

##### Habitat

Habitat importance: Major Importance

Habitats: 7.1. Caves and Subterranean Habitats (non-aquatic) - Caves

#### Habitat

Habitat importance: Major Importance

Habitats: 7.1. Caves and Subterranean Habitats (non-aquatic) - Caves

#### Ecology

Size: 5 mm

Generation length (yr): 1

Dependency of single sp?: No

Ecology and traits (narrative): This is a troglobitic species with low dispersal ability. It is known to feed on roots of the above-cave vegetation. It only occurs in six lava tubes in Pico Island, where it is restricted to the deep dark cave zone (Hoch 1991).

#### Threats

Justification for threats: This species is threatened by above-cave deforestation which will reduce the amount of roots suitable as food resources. The recent trend in removing exotic and native trees and shrubs for wine fields can be a major threat. Increasing pesticides in neighbouring agricultural land are also a threat. However, there are also several future potential threats: climatic changes will impact many habitats in the Azores (see Ferreira et al. 2016) and this can change the conditions inside the caves; change in the road infrastructure around the caves; potential human recreational activities with disturbance caused by radical cave visitation.

##### Threats

Threat type: Ongoing

Threats: 2.3. Agriculture & aquaculture - Livestock farming & ranching9.3. Pollution - Agricultural & forestry effluents

##### Threats

Threat type: Future

Threats: 4.1. Transportation & service corridors - Roads & railroads6.1. Human intrusions & disturbance - Recreational activities11.2. Climate change & severe weather - Droughts11.3. Climate change & severe weather - Temperature extremes

#### Threats

Threat type: Ongoing

Threats: 2.3. Agriculture & aquaculture - Livestock farming & ranching9.3. Pollution - Agricultural & forestry effluents

#### Threats

Threat type: Future

Threats: 4.1. Transportation & service corridors - Roads & railroads6.1. Human intrusions & disturbance - Recreational activities11.2. Climate change & severe weather - Droughts11.3. Climate change & severe weather - Temperature extremes

#### Conservation

Justification for conservation actions: This species is not protected by law in the Azores, but part of its distribution is included in protected areas namely in a protected landscape area (Furna dos Montanheiros inside de Pico Natural Park) and a natural monument (Gruta das Torres). Degraded habitats should be restored and a strategy needs to be developed to address the future threat by climate change that may change the vegetation cover of caves. A habitat management plan with associated education outreach initiatives is needed and anticipated to be developed during the coming years. It is necessary to establish a monitoring plan for the invertebrate community in the habitat in order to contribute to the conservation of this species.

##### Conservation actions

Conservation action type: Needed

Conservation actions: 2.1. Land/water management - Site/area management2.3. Land/water management - Habitat & natural process restoration5.4. Law & policy - Compliance and enforcement4.1. Education & awareness - Formal education4.3. Education & awareness - Awareness & communications

#### Conservation actions

Conservation action type: Needed

Conservation actions: 2.1. Land/water management - Site/area management2.3. Land/water management - Habitat & natural process restoration5.4. Law & policy - Compliance and enforcement4.1. Education & awareness - Formal education4.3. Education & awareness - Awareness & communications

#### Other

Justification for use and trade: Species not utilised

Justification for ecosystem services : Insufficient information available.

##### Use and trade

Use type: International

##### Ecosystem services

Ecosystem service type: Less important

##### Research needed

Research needed: 1.2. Research - Population size, distribution & trends1.3. Research - Life history & ecology2.2. Conservation Planning - Area-based Management Plan3.1. Monitoring - Population trends3.4. Monitoring - Habitat trends

Justification for research needed: Further research is needed into its ecology and life history in order to find extant specimens in additional caves. A species conservation plan and a management plan would improve the survival chances of this species for the future.

#### Use and trade

Use type: International

#### Ecosystem services

Ecosystem service type: Less important

#### Research needed

Research needed: 1.2. Research - Population size, distribution & trends1.3. Research - Life history & ecology2.2. Conservation Planning - Area-based Management Plan3.1. Monitoring - Population trends3.4. Monitoring - Habitat trends

Justification for research needed: Further research is needed into its ecology and life history in order to find extant specimens in additional caves. A species conservation plan and a management plan would improve the survival chances of this species for the future.

#### Viability analysis

### Thalassophilus azoricus

#### Species information

Scientific name: Thalassophilus azoricus

Species authority: Oromí & Borges, 1991

Common names: Cave ground beetle (English); Carocho cavernícola (Portuguese)

Kingdom: Animalia

Phylum: Arthropoda

Class: Insecta

Order: Coleoptera

Family: Carabidae

**Figure 4.**
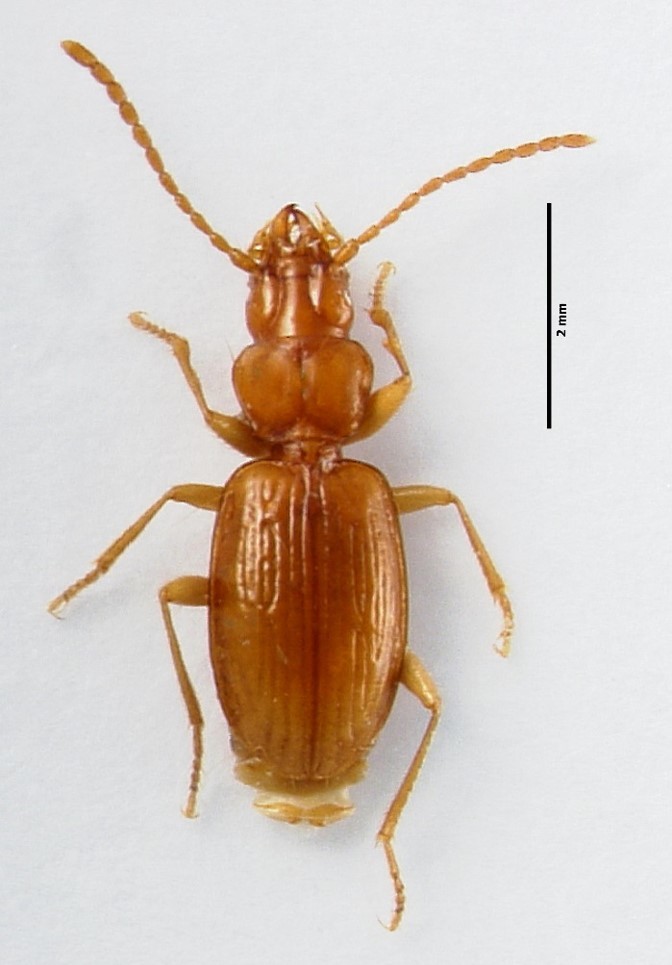
*Thalassophilus azoricus* (Oromí & Borges, 1991) from São Miguel island (Azores, Portugal) (Credit: Enésima Mendonça, Azorean Biodiversity Group, cE3c).

Figure(s) or Photo(s): Fig. 4

Region for assessment: Global

#### Editor & Reviewers

##### Reviewers

Reviewers: Anja Danielczak

##### Editor

Editor: Axel Hochkirch

#### Reviewers

Reviewers: Anja Danielczak

#### Editor

Editor: Axel Hochkirch

#### Geographic range

Biogeographic realm: Palearctic

Countries: Portugal

Map of records (Google Earth): Suppl. material 8

Basis of EOO and AOO: Observed

Basis (narrative): The extent of occurrence (EOO) is 8 km^2^ and the maximum estimated area of occupancy (AOO) is 8 km^2^

Min Elevation/Depth (m): 0

Max Elevation/Depth (m): 15

Range description: *Thalassophilus azoricus* is an endemic species from a single island, S. Miguel (Azores, Portugal) (Borges et al. 2010b) and known only from a single lava-tube cave, Gruta da Água de Pau (Oromí and Borges 1991).

#### New occurrences

#### Extent of occurrence

EOO (km2): 8

Trend: Stable

Justification for trend: This species occurs only in a single lava-tube cave in S. Miguel Island (Gruta de Água de Pau) (Oromí and Borges 1991). The value of EOO is an overestimation given that the EOO value is based on cave extent and not on the actual area ocuppied by the species which might be smaller.

Causes ceased?: Unknown

Causes understood?: Yes

Causes reversible?: Unknown

Extreme fluctuations?: No

#### Area of occupancy

Trend: Stable

Justification for trend: This species occurs only in single cave in S. Miguel island (Gruta de Água de Pau). The value of AOO given is an overestimation given that the AOO value is based on cave spatial occupancy, but the species in question might be restricted to a smaller area within the lava tube.

Causes ceased?: Unknown

Causes understood?: Yes

Causes reversible?: Unknown

Extreme fluctuations?: No

AOO (km2): 8

#### Locations

Number of locations: 1

Justification for number of locations: This species occurs only in single cave of S. Miguel island (Gruta de Água de Pau), that is under several threats, namely: i) residential and commercial development in coastal areas; ii) cave visitation by tourists and coastal recreational activities; iii) agriculture activities with cattle pollution; iv) creation of roads or coastal tracks; v) domestic water management.

Trend: Stable

Justification for trend: Only naturally present in a single location. Several caves in this island have been sampled for cave fauna (Borges et al. 2012) and this species has never been found at any other cave, meaning the current trend in number of locations is probably stable despite the impending threats.

Extreme fluctuations?: No

#### Population

Number of individuals: Unknown

Trend: Decline (inferred)

Justification for trend: The species is very rare and only known from a single population. A continuing decline in the number of mature individuals is inferred from the ongoing cave habitat degradation due to pasture cattle pollution and pollution resulting from fertiliser use.

Basis for decline: (c) a decline in area of occupancy, extent of occurrence and/or quality of habitat(d) actual or potential levels of exploitation

Causes ceased?: No

Causes understood?: Yes

Causes reversible?: Unknown

Extreme fluctuations?: No

Population Information (Narrative): Occurs naturally in a single cave under current and potential future threats, that include tourist visitation, cattle pollution and climate change. This is believed to be leading to a decrease in population numbers.

#### Subpopulations

Trend: Stable

Justification for trend: Only one subpopulation historically known.

Extreme fluctuations?: No

Severe fragmentation?: No

#### Habitat

System: Terrestrial

Habitat specialist: Yes

Habitat (narrative): This species occurs in a volcanic cave (a lava tube only 15 m above the sea level and covered by some 70 m of overburden) in the S. Miguel island (Gruta de Água de Pau) (Oromí and Borges 1991). The cave is small (323 m).

Trend in extent, area or quality?: Decline (inferred)

Justification for trend: The habitat is under several current and future threats (visitation, cattle pollution and Climate Change) (see more details below).

##### Habitat

Habitat importance: Major Importance

Habitats: 7.1. Caves and Subterranean Habitats (non-aquatic) - Caves

#### Habitat

Habitat importance: Major Importance

Habitats: 7.1. Caves and Subterranean Habitats (non-aquatic) - Caves

#### Ecology

Size: 2.98-3.42 mm

Generation length (yr): 1

Dependency of single sp?: No

Ecology and traits (narrative): This is a troglobiont species with considerable eye reduction, depigmentation and appendage elongation (Oromí and Borges 1991). The known specimens were captured under stones in humid sections of the cave.

#### Threats

Justification for threats: The main threats to this species are: i) residential and commercial development in coastal areas; ii) cave visitation by tourists and coastal recreational activities, promoting disturbance; iii) agriculture activities with cattle pollution; iv) creation of roads or coastal tracks; v) domestic water management; and vi) future climatic changes are expected to have impacts on coastal habitats (Ferreira et al. 2016).

##### Threats

Threat type: Ongoing

Threats: 1.3. Residential & commercial development - Tourism & recreation areas2.1.2. Agriculture & aquaculture - Annual & perennial non-timber crops - Small-holder farming6.1. Human intrusions & disturbance - Recreational activities7.2.1. Natural system modifications - Dams & water management/use - Abstraction of surface water (domestic use)9.1.2. Pollution - Domestic & urban waste water - Run-off

##### Threats

Threat type: Future

Threats: 4.1. Transportation & service corridors - Roads & railroads11.1. Climate change & severe weather - Habitat shifting & alteration11.4. Climate change & severe weather - Storms & flooding

#### Threats

Threat type: Ongoing

Threats: 1.3. Residential & commercial development - Tourism & recreation areas2.1.2. Agriculture & aquaculture - Annual & perennial non-timber crops - Small-holder farming6.1. Human intrusions & disturbance - Recreational activities7.2.1. Natural system modifications - Dams & water management/use - Abstraction of surface water (domestic use)9.1.2. Pollution - Domestic & urban waste water - Run-off

#### Threats

Threat type: Future

Threats: 4.1. Transportation & service corridors - Roads & railroads11.1. Climate change & severe weather - Habitat shifting & alteration11.4. Climate change & severe weather - Storms & flooding

#### Conservation

Justification for conservation actions: The species is protected by regional law (RAA 2012). Its habitat is in a regionally protected area (Natural Park of S. Miguel). We suggest, as a future measure of conservation, the creation of a fence in the entrance to the cave.

##### Conservation actions

Conservation action type: In Place

Conservation actions: 2. Land/water management

##### Conservation actions

Conservation action type: Needed

Conservation actions: 2.1. Land/water management - Site/area management4.3. Education & awareness - Awareness & communications

##### Conservation actions

Conservation action type: In Place

#### Conservation actions

Conservation action type: In Place

Conservation actions: 2. Land/water management

#### Conservation actions

Conservation action type: Needed

Conservation actions: 2.1. Land/water management - Site/area management4.3. Education & awareness - Awareness & communications

#### Conservation actions

Conservation action type: In Place

#### Other

Justification for use and trade: Species not utilised

Justification for ecosystem services : Insufficient Information Available

##### Use and trade

Use type: International

##### Ecosystem services

Ecosystem service type: Less important

##### Research needed

Research needed: 1.2. Research - Population size, distribution & trends1.3. Research - Life history & ecology2.2. Conservation Planning - Area-based Management Plan3.1. Monitoring - Population trends3.4. Monitoring - Habitat trends

Justification for research needed: Additional research is needed in order to determine the levels of population size as well as its ecology and life history. It is also necessary to establish a monitoring plan for the invertebrate community in the habitat in order to contribute to the conservation of this species.

#### Use and trade

Use type: International

#### Ecosystem services

Ecosystem service type: Less important

#### Research needed

Research needed: 1.2. Research - Population size, distribution & trends1.3. Research - Life history & ecology2.2. Conservation Planning - Area-based Management Plan3.1. Monitoring - Population trends3.4. Monitoring - Habitat trends

Justification for research needed: Additional research is needed in order to determine the levels of population size as well as its ecology and life history. It is also necessary to establish a monitoring plan for the invertebrate community in the habitat in order to contribute to the conservation of this species.

#### Viability analysis

### Trechus isabelae

#### Species information

Scientific name: Trechus isabelae

Species authority: Borges & Serrano, 2007

Common names: Cave ground-beetle (English); Carocho cavernícola (Portuguese)

Kingdom: Animalia

Phylum: Arthropoda

Class: Insecta

Order: Coleoptera

Family: Carabidae

**Figure 5.**
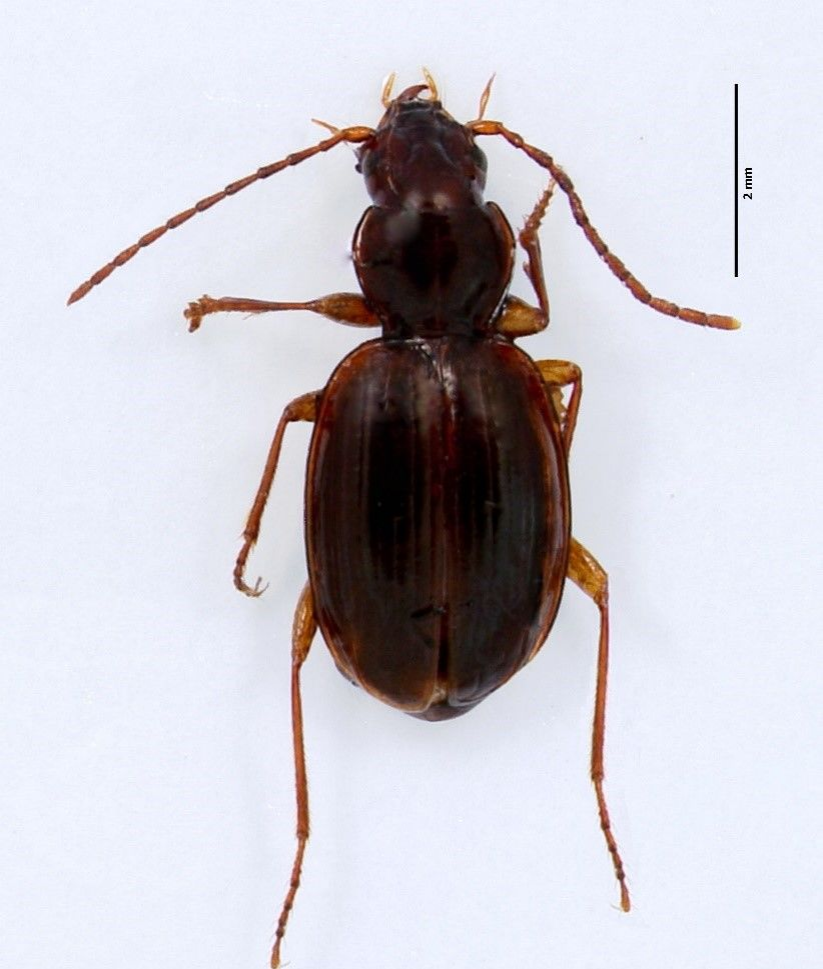
*Trechus isabelae* (Borges & Serrano, 2007) from São Jorge island (Azores, Portugal) (Credit: Paulo A.V. Borges).

Figure(s) or Photo(s): Fig. 5

Region for assessment: Global

#### Editor & Reviewers

##### Reviewers

Reviewers: Anja Danielczak

##### Editor

Editor: Axel Hochkirch

#### Reviewers

Reviewers: Anja Danielczak

#### Editor

Editor: Axel Hochkirch

#### Geographic range

Biogeographic realm: Palearctic

Countries: Portugal

Map of records (Google Earth): Suppl. material 9

Basis of EOO and AOO: Observed

Basis (narrative): The extent of occurrence (EOO) is 4 km^2^ and the maximum estimated area of occupancy (AOO) is 4 km^2^.

Min Elevation/Depth (m): 1000

Max Elevation/Depth (m): 1000

Range description: *Trechus isabelae* is a single island endemic cave-adapted species, restricted to S. Jorge (Azores, Portugal) (Borges et al. 2010b), known from a single cave, the volcanic pit Algar do Morro Pelado (Borges et al. 2007) located within the regionally protected area of Pico da Esperança e Planalto Central.

#### New occurrences

#### Extent of occurrence

EOO (km2): 4

Trend: Stable

Justification for trend: This species occurs only in a cave of S. Jorge island (Algar do Morro Pelado). The value of EOO is an overestimation given that the EOO value is restricted to cave size.

Causes ceased?: Yes

Causes understood?: Yes

Causes reversible?: Yes

Extreme fluctuations?: Unknown

#### Area of occupancy

Trend: Stable

Justification for trend: This species occurs only in a cave of S. Jorge island (Algar do Morro Pelado). The value of AOO is an overestimation given that the AOO value is restricted to cave size.

Causes ceased?: Yes

Causes understood?: Yes

Causes reversible?: Yes

Extreme fluctuations?: Unknown

AOO (km2): 4

#### Locations

Number of locations: 1

Justification for number of locations: This species occurs only in a cave of S. Jorge island (Algar do Morro Pelado) that, in spite of the fact that it has no current known threats, can be under future severe threats (see below).

Trend: Stable

Justification for trend: The single location is the full known historical range. Several caves in this island have been sampled for cave fauna (Borges et al. 2012) and this species has never been found at any other cave, possibly due to its being isolated in Algar do Morro Pelado volcanic region, meaning the current trend in number of locations is probably stable despite the impending future threats.

Extreme fluctuations?: Unknown

#### Population

Number of individuals: Unknown

Trend: Stable

Justification for trend: The species is very rare and only known from a single subpopulation. The number of individuals caught when the species was discovered was small when compared with other *Trechus* Azorean species more abundant in traps (e.g. *T. terceiranus* and *T picoensis*). The cave where the species occurs is located in a protected area and of difficult access and we assume there is no current impact for the population.

Causes ceased?: Yes

Causes understood?: Yes

Causes reversible?: Yes

Extreme fluctuations?: Unknown

#### Subpopulations

Trend: Stable

Justification for trend: Only one subpopulation historically known.

Extreme fluctuations?: No

Severe fragmentation?: No

#### Habitat

System: Terrestrial

Habitat specialist: Yes

Habitat (narrative): This species occurs in a volcanic pit (Algar do Morro Pelado, S. Jorge island) of great dimensions (140 m deep) located at 1000 m asl. The surrounding area consists of natural grassland (Borges et al. 2007).

Trend in extent, area or quality?: Stable

Justification for trend: The habitat is pristine and well preserved.

##### Habitat

Habitat importance: Major Importance

Habitats: 7.1. Caves and Subterranean Habitats (non-aquatic) - Caves

#### Habitat

Habitat importance: Major Importance

Habitats: 7.1. Caves and Subterranean Habitats (non-aquatic) - Caves

#### Ecology

Size: 4.78 mm

Generation length (yr): 1

Dependency of single sp?: No

Ecology and traits (narrative): This is a cavernicolous (i.e. a troglobitic species) predator and/or saprophagous species. The known specimens were captured only in pitfall traps.

#### Threats

Justification for threats: No threats are currently available, since the volcanic pit is located in an inaccessible area and the area is protected. However, there are several future potential threats: climatic changes will impact many habitats in Azores (see Ferreira et al. 2016) and this can change the conditions inside the volcanic pit; change in the road infrastructure around the cave; potential human recreational activities with radical cave visitation; reforestation of the area with exotic trees with unknown impact and geological events (volcanic activity, earthquakes and landslides).

##### Threats

Threat type: Future

Threats: 2.2.2. Agriculture & aquaculture - Wood & pulp plantations - Agro-industry plantations4.1. Transportation & service corridors - Roads & railroads6.1. Human intrusions & disturbance - Recreational activities10.1. Geological events - Volcanoes10.2. Geological events - Earthquakes/tsunamis10.3. Geological events - Avalanches/landslides11.1. Climate change & severe weather - Habitat shifting & alteration11.2. Climate change & severe weather - Droughts

#### Threats

Threat type: Future

Threats: 2.2.2. Agriculture & aquaculture - Wood & pulp plantations - Agro-industry plantations4.1. Transportation & service corridors - Roads & railroads6.1. Human intrusions & disturbance - Recreational activities10.1. Geological events - Volcanoes10.2. Geological events - Earthquakes/tsunamis10.3. Geological events - Avalanches/landslides11.1. Climate change & severe weather - Habitat shifting & alteration11.2. Climate change & severe weather - Droughts

#### Conservation

Justification for conservation actions: The species is protected by regional law (RAA 2012). Its habitat is in a regionally protected area (Natural Park of S. Jorge, within the regionally protected area of Pico da Esperança e Planalto Central). It is necessary to establish a monitoring plan for the invertebrate community in the habitat in order to contribute to the conservation of this species. A habitat management plan is needed and is anticipated to be developed during the coming years.

##### Conservation actions

Conservation action type: In Place

Conservation actions: 1.1. Land/water protection - Site/area protection2.1. Land/water management - Site/area management

##### Conservation actions

Conservation action type: Needed

Conservation actions: 2.1. Land/water management - Site/area management4. Education & awareness5.4.3. Law & policy - Compliance and enforcement - Sub-national level

#### Conservation actions

Conservation action type: In Place

Conservation actions: 1.1. Land/water protection - Site/area protection2.1. Land/water management - Site/area management

#### Conservation actions

Conservation action type: Needed

Conservation actions: 2.1. Land/water management - Site/area management4. Education & awareness5.4.3. Law & policy - Compliance and enforcement - Sub-national level

#### Other

Justification for use and trade: The species is not utilised.

Justification for ecosystem services : Insufficient information available.

##### Use and trade

Use type: International

##### Ecosystem services

Ecosystem service type: Less important

##### Research needed

Research needed: 1.2. Research - Population size, distribution & trends1.3. Research - Life history & ecology2.2. Conservation Planning - Area-based Management Plan3.1. Monitoring - Population trends3.4. Monitoring - Habitat trends

Justification for research needed: Additional research is needed in order to determine the levels of population size as well as its ecology and life history. A monitoring plan is also necessary for the invertebrate community in the habitat in order to contribute to the conservation of this species.

#### Use and trade

Use type: International

#### Ecosystem services

Ecosystem service type: Less important

#### Research needed

Research needed: 1.2. Research - Population size, distribution & trends1.3. Research - Life history & ecology2.2. Conservation Planning - Area-based Management Plan3.1. Monitoring - Population trends3.4. Monitoring - Habitat trends

Justification for research needed: Additional research is needed in order to determine the levels of population size as well as its ecology and life history. A monitoring plan is also necessary for the invertebrate community in the habitat in order to contribute to the conservation of this species.

#### Viability analysis

### Trechus jorgensis

#### Species information

Scientific name: Trechus jorgensis

Species authority: Oromí & Borges, 1991

Common names: Cave ground-beetle (English) ; Carocho cavernícola (Portuguese)

Kingdom: Animalia

Phylum: Arthropoda

Class: Insecta

Order: Coleoptera

Family: Carabidae

**Figure 6.**
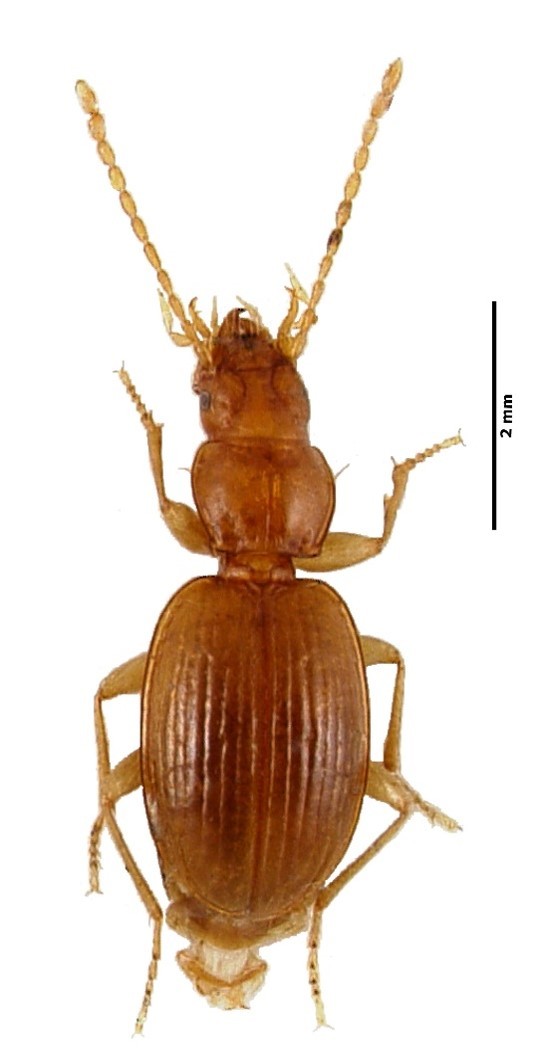
*Trechus jorgensis* (Oromí & Borges, 1991) from São Jorge island (Azores, Portugal) (Credit: Enésima Mendonça, Azorean Biodiversity Group, cE3c).

Figure(s) or Photo(s): Fig. 6

Region for assessment: Global

#### Editor & Reviewers

##### Reviewers

Reviewers: Anja Danielczak

##### Editor

Editor: Axel Hochkirch

#### Reviewers

Reviewers: Anja Danielczak

#### Editor

Editor: Axel Hochkirch

#### Geographic range

Biogeographic realm: Palearctic

Countries: Portugal

Map of records (Google Earth): Suppl. material 10

Basis of EOO and AOO: Observed

Basis (narrative): The extent of occurrence (EOO) is 4 km^2^ and the maximum estimated area of occupancy (AOO) is 4 km^2^.

Min Elevation/Depth (m): 385

Max Elevation/Depth (m): 385

Range description: *Trechus jorgensis* is an endemic cave-adapted species known from a single island, S. Jorge (Azores, Portugal) (Borges et al. 2010b) and occurs in a single cave, the volcanic pit of Algar das Bocas do Fogo (Oromí and Borges 1991).

#### New occurrences

#### Extent of occurrence

EOO (km2): 4

Trend: Decline (inferred)

Justification for trend: This species occurs only in a cave of S. Jorge island (Algar das Bocas do Fogo). The species is threatened due to the loss of habitat quality, since the volcanic pit has been used as a dump site. In addition, there is ongoing destruction of the surrounding habitat due to the spread of invasive plants.

Causes ceased?: No

Causes understood?: Yes

Causes reversible?: Yes

Extreme fluctuations?: Unknown

#### Area of occupancy

Trend: Decline (inferred)

Justification for trend: This species occurs only in a cave of S. Jorge island (Algar das Bocas do Fogo). The species is threatened due to the loss of habitat quality, since the volcanic pit has been used as a dump site. In addition, there is ongoing destruction of the surrounding habitat due to the spread of invasive plants.

Causes ceased?: No

Causes understood?: Yes

Causes reversible?: Yes

Extreme fluctuations?: Unknown

AOO (km2): 4

#### Locations

Number of locations: 1

Justification for number of locations: This species occurs only in a cave of S. Jorge island (Algar das Bocas do Fogo), which is currently under serious threat.

Trend: Stable

Justification for trend: The single location is the full known historical range.

Extreme fluctuations?: Unknown

#### Population

Number of individuals: Unknown

Trend: Decline (inferred)

Justification for trend: The species is very rare and only known from a single subpopulation. In all the sampling occasions, only a small number of specimens were collected. The area surrounding the cave is heavily impacted by human activities and used as a dump area.

Basis for decline: (c) a decline in area of occupancy, extent of occurrence and/or quality of habitat

Causes ceased?: No

Causes understood?: Yes

Causes reversible?: Unknown

Extreme fluctuations?: Unknown

#### Subpopulations

Trend: Stable

Justification for trend: Only one subpopulation historically known.

Extreme fluctuations?: No

Severe fragmentation?: No

#### Habitat

System: Terrestrial

Habitat specialist: Yes

Habitat (narrative): This species occurs in a 50 m deep volcanic pit (Algar das Bocas do Fogo, S. Jorge island), whose internal vault is in penumbra (not complete darkness). The surrounding area consists of exotic plantations of *Pittosporum undulatum* (Oromí and Borges 1991).

Trend in extent, area or quality?: Decline (observed)

Justification for trend: The habitat is under severe destruction by being located in a dump area.

##### Habitat

Habitat importance: Major Importance

Habitats: 7.1. Caves and Subterranean Habitats (non-aquatic) - Caves

#### Habitat

Habitat importance: Major Importance

Habitats: 7.1. Caves and Subterranean Habitats (non-aquatic) - Caves

#### Ecology

Size: 3.05 mm

Generation length (yr): 1

Dependency of single sp?: No

Ecology and traits (narrative): This is a cavernicolous (i.e. a troglobitic species) predator and/or saprophagous species. The known specimens were captured only in pitfall traps.

#### Threats

Justification for threats: The main current threats to this species are the loss of habitat quality, as the volcanic pit has been used as a dump site, as well as the destruction of the surrounding habitat by invasive plants. However, there are several future potential threats: climatic changes will impact many habitats in Azores (see Ferreira et al. 2016) and this can change the conditions inside the volcanic pit; change in the road and urban infrastructure around the cave.

##### Threats

Threat type: Ongoing

Threats: 2.1.2. Agriculture & aquaculture - Annual & perennial non-timber crops - Small-holder farming2.2.2. Agriculture & aquaculture - Wood & pulp plantations - Agro-industry plantations4.1. Transportation & service corridors - Roads & railroads6.1. Human intrusions & disturbance - Recreational activities7.2.1. Natural system modifications - Dams & water management/use - Abstraction of surface water (domestic use)8.1.1. Invasive and other problematic species, genes & diseases - Invasive non-native/alien species/diseases - Unspecified species9.1.2. Pollution - Domestic & urban waste water - Run-off

##### Threats

Threat type: Future

Threats: 1.2. Residential & commercial development - Commercial & industrial areas11.1. Climate change & severe weather - Habitat shifting & alteration11.2. Climate change & severe weather - Droughts

#### Threats

Threat type: Ongoing

Threats: 2.1.2. Agriculture & aquaculture - Annual & perennial non-timber crops - Small-holder farming2.2.2. Agriculture & aquaculture - Wood & pulp plantations - Agro-industry plantations4.1. Transportation & service corridors - Roads & railroads6.1. Human intrusions & disturbance - Recreational activities7.2.1. Natural system modifications - Dams & water management/use - Abstraction of surface water (domestic use)8.1.1. Invasive and other problematic species, genes & diseases - Invasive non-native/alien species/diseases - Unspecified species9.1.2. Pollution - Domestic & urban waste water - Run-off

#### Threats

Threat type: Future

Threats: 1.2. Residential & commercial development - Commercial & industrial areas11.1. Climate change & severe weather - Habitat shifting & alteration11.2. Climate change & severe weather - Droughts

#### Conservation

Justification for conservation actions: Although the species is protected by regional law (RAA 2012), the cave where it occurs is, however, not protected. It is necessary to establish a monitoring plan for the invertebrate community in the habitat in order to contribute to the conservation of this species. We suggest as future measures for conservation: a scoping mission to determine thecurrent status of the cave habitat throughout the full extent of the cave; restoring habitat by cleaning the trash from the cave; the creation of a fence surrounding the top of the pit. A habitat management plan is needed and is anticipated to be developed during the coming years.

##### Conservation actions

Conservation action type: Needed

Conservation actions: 1.1. Land/water protection - Site/area protection2.1. Land/water management - Site/area management5.4.3. Law & policy - Compliance and enforcement - Sub-national level4.1. Education & awareness - Formal education

#### Conservation actions

Conservation action type: Needed

Conservation actions: 1.1. Land/water protection - Site/area protection2.1. Land/water management - Site/area management5.4.3. Law & policy - Compliance and enforcement - Sub-national level4.1. Education & awareness - Formal education

#### Other

Justification for use and trade: The species is not utilised.

Justification for ecosystem services : Insufficient information available.

##### Use and trade

Use type: International

##### Ecosystem services

Ecosystem service type: Less important

##### Research needed

Research needed: 1.2. Research - Population size, distribution & trends1.3. Research - Life history & ecology2.2. Conservation Planning - Area-based Management Plan3.1. Monitoring - Population trends3.4. Monitoring - Habitat trends

Justification for research needed: Additional research is needed in order to determine the levels of population size as well as its ecology and life history. A monitoring plan is also necessary for the invertebrate community in the habitat in order to contribute to the conservation of this species.

#### Use and trade

Use type: International

#### Ecosystem services

Ecosystem service type: Less important

#### Research needed

Research needed: 1.2. Research - Population size, distribution & trends1.3. Research - Life history & ecology2.2. Conservation Planning - Area-based Management Plan3.1. Monitoring - Population trends3.4. Monitoring - Habitat trends

Justification for research needed: Additional research is needed in order to determine the levels of population size as well as its ecology and life history. A monitoring plan is also necessary for the invertebrate community in the habitat in order to contribute to the conservation of this species.

#### Viability analysis

### Trechus montanheirorum

#### Species information

Scientific name: Trechus montanheirorum

Species authority: Oromí & Borges, 1991

Common names: Cave ground-beetle (English); Carocho cavernícola (Portuguese)

Kingdom: Animalia

Phylum: Arthropoda

Class: Insecta

Order: Coleoptera

Family: Carabidae

**Figure 7.**
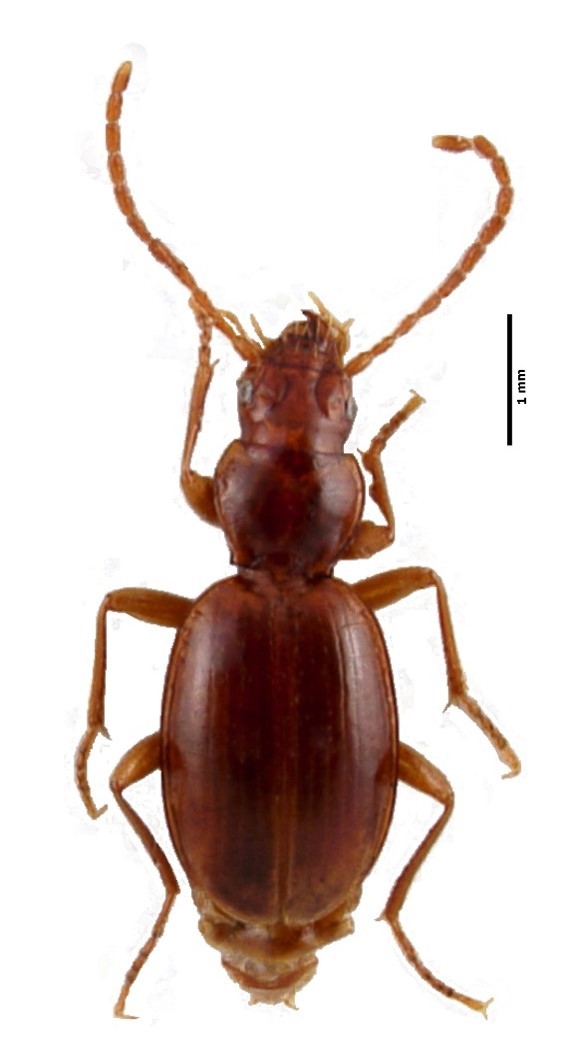
*Trechus montanheirorum* (Oromí & Borges, 1991) from Pico island (Azores, Portugal) (Credit: Enésima Mendonça, Azorean Biodiversity Group, cE3c).

Figure(s) or Photo(s): Fig. 7

Region for assessment: Global

#### Editor & Reviewers

##### Reviewers

Reviewers: Anja Danielczak

##### Editor

Editor: Axel Hochkirch

#### Reviewers

Reviewers: Anja Danielczak

#### Editor

Editor: Axel Hochkirch

#### Geographic range

Biogeographic realm: Palearctic

Countries: Portugal

Map of records (Google Earth): Suppl. material 11

Basis of EOO and AOO: Observed

Basis (narrative): The extent of occurrence (EOO) is 12 km^2^ and the maximum estimated area of occupancy (AOO) is 12 km^2^.

Min Elevation/Depth (m): 580

Max Elevation/Depth (m): 770

Range description: *Trechus montanheirorum* is an endemic cave-adapted species known from Pico (Azores, Portugal) (Borges et al. 2010b), occurring in only three lava tube caves (Furna de Frei Matias, Furna dos Montanheiros and Gruta dos Vimes) (Pereira et al. 2015).

#### New occurrences

#### Extent of occurrence

EOO (km2): 12

Trend: Decline (inferred)

Justification for trend: This species occurs in three caves of Pico island (Furna de Frei Matias, Furna dos Montanheiros and Gruta dos Vimes). No decrease in EOO has been registered, but it is inferred from decline in the habitat quality in two of the caves.

Causes ceased?: No

Causes understood?: Yes

Causes reversible?: Yes

Extreme fluctuations?: Unknown

#### Area of occupancy

Trend: Decline (inferred)

Justification for trend: This species occurs in three caves of Pico island (Furna de Frei Matias, Furna dos Montanheiros and Gruta dos Vimes). No decrease in AOO has been registered, but it is inferred from decline in habitat quality in two of the caves.

Causes ceased?: No

Causes understood?: Yes

Causes reversible?: Yes

Extreme fluctuations?: Unknown

AOO (km2): 12

#### Locations

Number of locations: 3

Justification for number of locations: This species occurs in three caves of Pico island (Furna de Frei Matias, Furna dos Montanheiros and Gruta dos Vimes). In one of the caves (Furna de Frei Matias), the species is threatened by uncontrolled visits and, in the other two, by dairy cattle pollution.

Trend: Stable

Justification for trend: The current trend in number of locations is probably stable despite the impending threats.

Extreme fluctuations?: Unknown

#### Population

Number of individuals: Unknown

Trend: Decline (inferred)

Justification for trend: The area surrounding one of the the caves (Furna dos Montanheiros) is relatively well protected, but the area surrounding the other two caves is more disturbed. Therefore, we assume relatively few impacts for the population but infer some decrease in density of individuals in at least two of the caves. However, tourism recreational activities could be a problem as well as agriculture management in two of the caves.

Basis for decline: (c) a decline in area of occupancy, extent of occurrence and/or quality of habitat

Causes ceased?: No

Causes understood?: Yes

Causes reversible?: Unknown

Extreme fluctuations?: Unknown

Population Information (Narrative): The species is relatively abundant in at least one of the caves (Furna dos Montanheiros). The area surrounding one of the caves (Furna dos Montanheiros) is relatively well protected, but the area surrounding the other two caves is more disturbed.

#### Subpopulations

Trend: Decline (inferred)

Justification for trend: In at least two of the subpopulations, the threats are sufficient for placing the subpopulations at a risk of extinction (Furna do Frei Matias and Gruta dos Vimes).

Extreme fluctuations?: No

Severe fragmentation?: No

#### Habitat

System: Terrestrial

Habitat specialist: Yes

Habitat (narrative): This species occurs in three caves of Pico island (Furna de Frei Matias, Furna dos Montanheiros and Gruta dos Vimes). This species has some ability to colonise the entrances to the caves, but no specimens were ever collected outside a cave (Oromí and Borges 1991, Amorim 2005).

Trend in extent, area or quality?: Decline (observed)

Justification for trend: There is already some impact from touristic visitation in at least one of the caves (Furna Frei Matias).

##### Habitat

Habitat importance: Major Importance

Habitats: 7.1. Caves and Subterranean Habitats (non-aquatic) - Caves

#### Habitat

Habitat importance: Major Importance

Habitats: 7.1. Caves and Subterranean Habitats (non-aquatic) - Caves

#### Ecology

Size: 4.27-4.88 mm

Generation length (yr): 1

Dependency of single sp?: No

Ecology and traits (narrative): This is a cavernicolous (i.e. a troglobitic species) predator and/or saprophagous species.

#### Threats

Justification for threats: The main current threats to this species are the loss of habitat quality, due to recreational cave visitation and the impact of pasture lands. In one of the caves (Furna de Frei Matias), the species is threatened by uncontrolled visits and, in the other two, by dairy cattle pollution. However, there are several future potential threats: climatic changes will impact many habitats in the Azores (see Ferreira et al. 2016) and this can change the conditions inside all three caves; change in the road infrastructure around the cave (particularly important to Furna dos Montanheiros that crosses a main road); potential human recreational activities with disturbance caused by radical cave visitation (particularly important to Furna de Frei Matias and Furna dos Montanheiros); reforestation of the area with exotic trees with unknown impact (important for all three caves).

##### Threats

Threat type: Ongoing

Threats: 1.3. Residential & commercial development - Tourism & recreation areas2.1.2. Agriculture & aquaculture - Annual & perennial non-timber crops - Small-holder farming2.2.2. Agriculture & aquaculture - Wood & pulp plantations - Agro-industry plantations6.1. Human intrusions & disturbance - Recreational activities10.2. Geological events - Earthquakes/tsunamis

##### Threats

Threat type: Future

Threats: 4.1. Transportation & service corridors - Roads & railroads11.1. Climate change & severe weather - Habitat shifting & alteration11.2. Climate change & severe weather - Droughts

#### Threats

Threat type: Ongoing

Threats: 1.3. Residential & commercial development - Tourism & recreation areas2.1.2. Agriculture & aquaculture - Annual & perennial non-timber crops - Small-holder farming2.2.2. Agriculture & aquaculture - Wood & pulp plantations - Agro-industry plantations6.1. Human intrusions & disturbance - Recreational activities10.2. Geological events - Earthquakes/tsunamis

#### Threats

Threat type: Future

Threats: 4.1. Transportation & service corridors - Roads & railroads11.1. Climate change & severe weather - Habitat shifting & alteration11.2. Climate change & severe weather - Droughts

#### Conservation

Justification for conservation actions: The species is protected by regional law (RAA 2012). Its habitat is in a regionally protected area (Natural Park of Pico), but only one of the three caves where this species occurs is within a regionally protected area. It is necessary to establish a monitoring plan for the invertebrate community in the cave habitat in order to contribute to the conservation of this species. We suggest, as a future measure of conservation, the fencing of the entrances to the caves where human intrusion and disturbance have been occurring. A habitat management plan is needed and is anticipated to be developed during the coming years.

##### Conservation actions

Conservation action type: In Place

Conservation actions: 1.1. Land/water protection - Site/area protection2.1. Land/water management - Site/area management

##### Conservation actions

Conservation action type: Needed

Conservation actions: 2.1. Land/water management - Site/area management5.4.3. Law & policy - Compliance and enforcement - Sub-national level4.1. Education & awareness - Formal education

#### Conservation actions

Conservation action type: In Place

Conservation actions: 1.1. Land/water protection - Site/area protection2.1. Land/water management - Site/area management

#### Conservation actions

Conservation action type: Needed

Conservation actions: 2.1. Land/water management - Site/area management5.4.3. Law & policy - Compliance and enforcement - Sub-national level4.1. Education & awareness - Formal education

#### Other

Justification for use and trade: The species is not utilised.

Justification for ecosystem services : Insufficient information available

##### Use and trade

Use type: International

##### Ecosystem services

Ecosystem service type: Less important

##### Research needed

Research needed: 1.2. Research - Population size, distribution & trends1.3. Research - Life history & ecology2.2. Conservation Planning - Area-based Management Plan3.1. Monitoring - Population trends3.4. Monitoring - Habitat trends

Justification for research needed: Additional research is needed in order to determine the levels of population size as well as its ecology and life history. It is also necessary to establish a monitoring plan for the invertebrate community in the habitat in order to contribute to the conservation of this species.

#### Use and trade

Use type: International

#### Ecosystem services

Ecosystem service type: Less important

#### Research needed

Research needed: 1.2. Research - Population size, distribution & trends1.3. Research - Life history & ecology2.2. Conservation Planning - Area-based Management Plan3.1. Monitoring - Population trends3.4. Monitoring - Habitat trends

Justification for research needed: Additional research is needed in order to determine the levels of population size as well as its ecology and life history. It is also necessary to establish a monitoring plan for the invertebrate community in the habitat in order to contribute to the conservation of this species.

#### Viability analysis

### Trechus oromii

#### Species information

Scientific name: Trechus oromii

Species authority: Borges, Serrano & Amorim, 2004

Common names: Cave ground-beetle (English); Carocho cavernícola (Portuguese)

Kingdom: Animalia

Phylum: Arthropoda

Class: Insecta

Order: Coleoptera

Family: Carabidae

**Figure 8.**
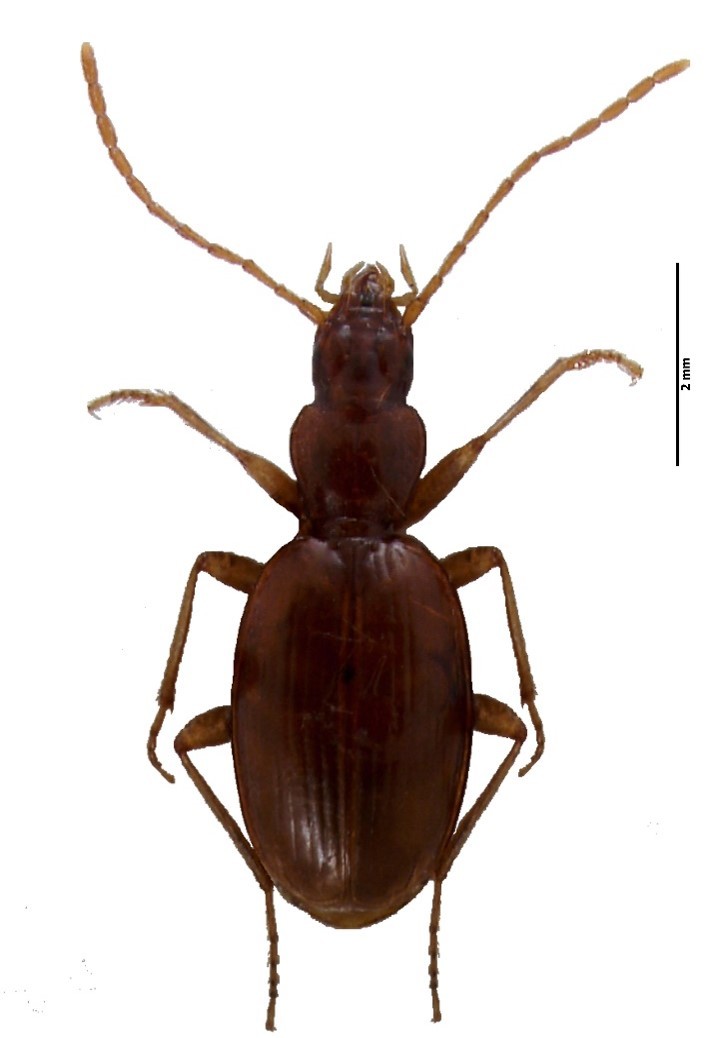
*Trechus oromii* (Borges, Serrano & Amorim, 2004) from Faial island (Azores, Portugal) (Credit: Enésima Mendonça, Azorean Biodiversity Group, cE3c).

Figure(s) or Photo(s): Fig. 8

Region for assessment: Global

#### Editor & Reviewers

##### Reviewers

Reviewers: Anja Danielczak

##### Editor

Editor: Axel Hochkirch

#### Reviewers

Reviewers: Anja Danielczak

#### Editor

Editor: Axel Hochkirch

#### Geographic range

Biogeographic realm: Palearctic

Countries: Portugal

Map of records (Google Earth): Suppl. material 12

Basis of EOO and AOO: Observed

Basis (narrative): The extent of occurrence (EOO) is 4 km^2^ and the maximum estimated area of occupancy (AOO) is 4 km^2^.

Min Elevation/Depth (m): 254

Max Elevation/Depth (m): 254

Range description: *Trechus oromii* is a cave-adapted endemic species known from Faial (Azores, Portugal) (Borges et al. 2010b), occurring in only one lava tube cave (Gruta do Parque do Capelo) (Borges et al. 2004).

#### New occurrences

#### Extent of occurrence

EOO (km2): 4

Trend: Decline (inferred)

Justification for trend: This species occurs only in a cave of Faial island (Gruta do Parque do Capelo). No decrease in EOO has been registered, but it is inferred from decline in the habitat quality.

Causes ceased?: No

Causes understood?: Yes

Causes reversible?: Yes

Extreme fluctuations?: Unknown

#### Area of occupancy

Trend: Decline (inferred)

Justification for trend: This species occurs only in a cave of Faial island (Gruta do Parque do Capelo). No decrease in AOO has been registered, but it is inferred from decline in the habitat quality.

Causes ceased?: No

Causes understood?: Yes

Causes reversible?: Yes

Extreme fluctuations?: Unknown

AOO (km2): 4

#### Locations

Number of locations: 1

Justification for number of locations: This species occurs only in a cave of Faial island (Gruta do Parque do Capelo) that is threatened by the loss of habitat quality, due to recreational cave visitation and to the impact of the management of the Nature Reserve & Recreational Park, where the cave is located.

Trend: Stable

Justification for trend: The single location is the full known historical range.

Extreme fluctuations?: Unknown

#### Population

Number of individuals: Unknown

Trend: Decline (estimated)

Justification for trend: The area surrounding the cave is heavily impacted by human disturbance. The cave entrance is located in a forest recreational park with several kind of activities and it is frequent to see some uncontrolled cave visitation.

Basis for decline: (c) a decline in area of occupancy, extent of occurrence and/or quality of habitat

Causes ceased?: No

Causes understood?: Yes

Causes reversible?: Unknown

Extreme fluctuations?: Unknown

Population Information (Narrative): The species is very rare and only known from a single subpopulation in Faial island.

#### Subpopulations

Trend: Stable

Justification for trend: Only one subpopulation historically known.

Extreme fluctuations?: No

Severe fragmentation?: No

#### Habitat

System: Terrestrial

Habitat specialist: Yes

Habitat (narrative): The species is very rare and only known from a single subpopulation in Faial island. The area surrounding the cave is heavily impacted by human disturbance.

Trend in extent, area or quality?: Decline (observed)

Justification for trend: There is already some impact from construction and human activity in the Forest Park, where it is located. Unfortunately, some uncontrolled cave visitation is frequently seen. In addition, there was a recent reorganisation of the park with some trees removed and new buildings and trails created with unknown impacts on the cave.

##### Habitat

Habitat importance: Major Importance

Habitats: 7.1. Caves and Subterranean Habitats (non-aquatic) - Caves

#### Habitat

Habitat importance: Major Importance

Habitats: 7.1. Caves and Subterranean Habitats (non-aquatic) - Caves

#### Ecology

Size: 4.64-4.82 mm

Generation length (yr): 1

Dependency of single sp?: No

Ecology and traits (narrative): This is a cavernicolous (i.e. a troglobitic species) predator and/or saprophagous species.

#### Threats

Justification for threats: The main current threats to this species are the loss of habitat quality, due to recreational cave visitation and the impact of the management of the Nature Reserve & Recreational Park, where the cave is located. Unfortunately, some uncontrolled cave visitation is frequently seen. In addition, there was a recent reorganisation of the park with some trees removed and new buildings and trails created with unknown impacts on the cave. However, there are several future potential threats: climatic changes will impact many habitats in the Azores (see Ferreira et al. 2016) and this can change the conditions inside the cave; change in the road infraestructure around the cave; potential human recreational activities with disturbance caused by radical cave visitation.

##### Threats

Threat type: Ongoing

Threats: 2.2.2. Agriculture & aquaculture - Wood & pulp plantations - Agro-industry plantations6.1. Human intrusions & disturbance - Recreational activities

##### Threats

Threat type: Future

Threats: 4. Transportation & service corridors11.1. Climate change & severe weather - Habitat shifting & alteration11.2. Climate change & severe weather - Droughts

#### Threats

Threat type: Ongoing

Threats: 2.2.2. Agriculture & aquaculture - Wood & pulp plantations - Agro-industry plantations6.1. Human intrusions & disturbance - Recreational activities

#### Threats

Threat type: Future

Threats: 4. Transportation & service corridors11.1. Climate change & severe weather - Habitat shifting & alteration11.2. Climate change & severe weather - Droughts

#### Conservation

Justification for conservation actions: The species is protected by regional law (RAA 2012). Its habitat is in a regionally protected area (Natural Park of Faial). It is necessary to establish a monitoring plan for the invertebrate community in the habitat in order to contribute to the conservation of this species. We suggest, as a future measure of conservation, fencing the entrances to the caves where human intrusion and disturbance have been occurring. A habitat management plan is needed and is anticipated to be developed during the coming years.

##### Conservation actions

Conservation action type: In Place

Conservation actions: 1.1. Land/water protection - Site/area protection2.1. Land/water management - Site/area management

##### Conservation actions

Conservation action type: Needed

Conservation actions: 2.1. Land/water management - Site/area management4. Education & awareness5.4.3. Law & policy - Compliance and enforcement - Sub-national level

#### Conservation actions

Conservation action type: In Place

Conservation actions: 1.1. Land/water protection - Site/area protection2.1. Land/water management - Site/area management

#### Conservation actions

Conservation action type: Needed

Conservation actions: 2.1. Land/water management - Site/area management4. Education & awareness5.4.3. Law & policy - Compliance and enforcement - Sub-national level

#### Other

Justification for use and trade: The species is not utilised.

Justification for ecosystem services : Insufficient information available.

##### Use and trade

Use type: International

##### Ecosystem services

Ecosystem service type: Less important

##### Research needed

Research needed: 1.2. Research - Population size, distribution & trends1.3. Research - Life history & ecology2.2. Conservation Planning - Area-based Management Plan3.1. Monitoring - Population trends3.4. Monitoring - Habitat trends

Justification for research needed: Additional research is needed in order to determine the levels of population size, as well as its ecology and life history. It is also necessary to establish a monitoring plan for the invertebrate community in the habitat in order to contribute to the conservation of this species.

#### Use and trade

Use type: International

#### Ecosystem services

Ecosystem service type: Less important

#### Research needed

Research needed: 1.2. Research - Population size, distribution & trends1.3. Research - Life history & ecology2.2. Conservation Planning - Area-based Management Plan3.1. Monitoring - Population trends3.4. Monitoring - Habitat trends

Justification for research needed: Additional research is needed in order to determine the levels of population size, as well as its ecology and life history. It is also necessary to establish a monitoring plan for the invertebrate community in the habitat in order to contribute to the conservation of this species.

#### Viability analysis

### Trechus pereirai

#### Species information

Scientific name: Trechus pereirai

Species authority: Borges, Serrano & Amorim, 2004

Common names: Cave ground-beetle (English); Carocho cavernícola (Portuguese)

Kingdom: Animalia

Phylum: Arthropoda

Class: Insecta

Order: Coleoptera

Family: Carabidae

**Figure 9.**
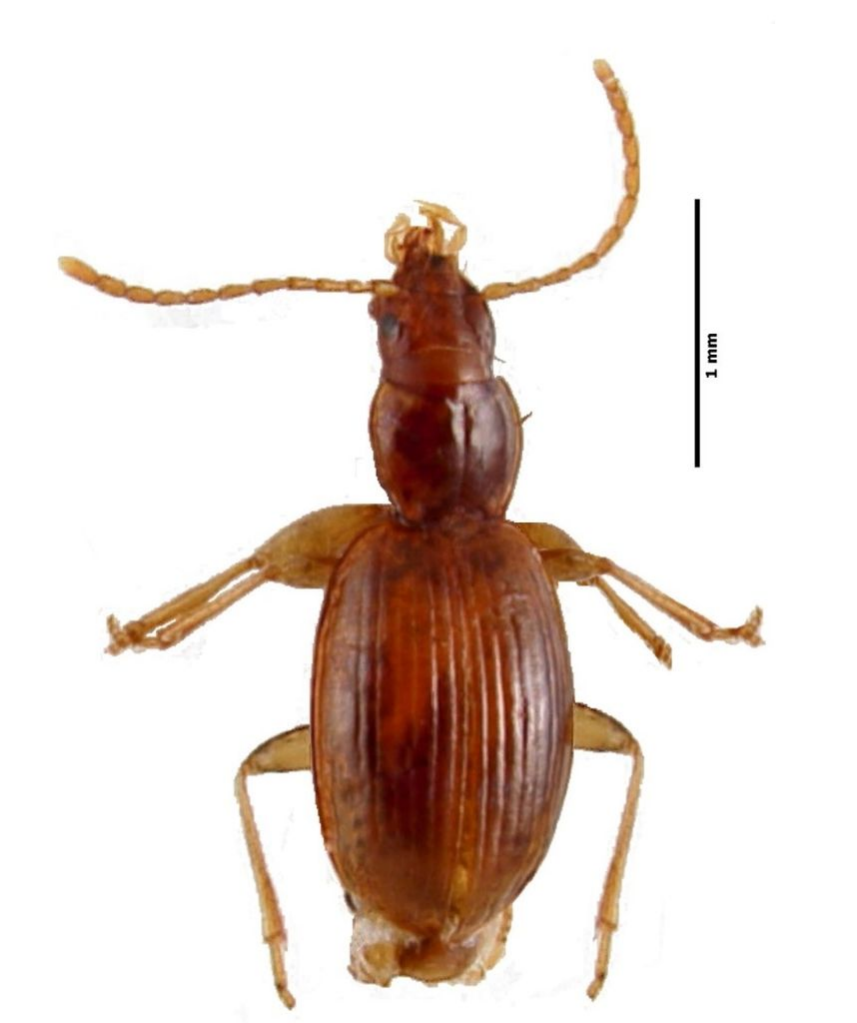
*Trechus pereirai* (Borges, Serrano & Amorim, 2004) from Pico island (Azores, Portugal) (Credit: Enésima Mendonça).

Figure(s) or Photo(s): Fig. 9

Region for assessment: Global

#### Editor & Reviewers

##### Reviewers

Reviewers: Anja Danielczak

##### Editor

Editor: Axel Hochkirch

#### Reviewers

Reviewers: Anja Danielczak

#### Editor

Editor: Axel Hochkirch

#### Geographic range

Biogeographic realm: Palearctic

Countries: Portugal

Map of records (Google Earth): Suppl. material 13

Basis of EOO and AOO: Observed

Basis (narrative): The extent of occurrence (EOO) is 8 km^2^ and the maximum estimated area of occupancy (AOO) is 8 km^2^.

Min Elevation/Depth (m): 180

Max Elevation/Depth (m): 200

Range description: *Trechus pereirai* is an endemic cave-adapted species known from a single island, Pico (Azores, Portugal) (Borges et al. 2010b), occurring in two lava-tube caves (Furna das Cabras II and Gruta da Ribeira do Fundo).

#### New occurrences

#### Extent of occurrence

EOO (km2): 8

Trend: Decline (inferred)

Justification for trend: The species occurs only in two lava-tube caves (Furna das Cabras II -200 m length; and Gruta da Ribeira do Fundo - 200 m length) on the island of Pico (Azores, Portugal). The value of EOO is an overestimation given that the EOO value is based on cave size and not on the actual area occupied by the species which might be smaller. No decrease in EOO has been registered but it is inferred from decline in the habitat quality.

Causes ceased?: No

Causes understood?: Yes

Causes reversible?: Yes

Extreme fluctuations?: Unknown

#### Area of occupancy

Trend: Decline (inferred)

Justification for trend: The species occurs only in two lava-tube caves (Furna das Cabras II and Gruta da Ribeira do Fundo) on the island of Pico (Azores, Portugal). The value of AOO given is an overestimation given that the AOO value is based on cave size, but the species in question might be restricted to a smaller area within the lava tube. No decrease in AOO has been registered, but it is inferred from decline in the habitat quality.

Causes ceased?: No

Causes understood?: Yes

Causes reversible?: Yes

Extreme fluctuations?: Unknown

AOO (km2): 8

#### Locations

Number of locations: 2

Justification for number of locations: The species occurs only in two lava-tube caves (Furna das Cabras II and Gruta da Ribeira do Fundo) on the island of Pico (Azores, Portugal) and which are currently under serious threat.

Trend: Stable

Justification for trend: The current trend in number of locations is probably stable despite the impending threats.

Extreme fluctuations?: No

#### Population

Number of individuals: Unknown

Trend: Decline (inferred)

Justification for trend: Species abundance may have decreased in one of the caves (Gruta da Ribeira do Fundo) as it has been used as a dump site up till recently; and the population in the other cave where this species occurs (Furna das Cabras II) may be negatively impacted since the area surrounding the cave is suitable for forest exploitation.

Basis for decline: (c) a decline in area of occupancy, extent of occurrence and/or quality of habitat

Causes ceased?: No

Causes understood?: Yes

Causes reversible?: Unknown

Extreme fluctuations?: Unknown

Population Information (Narrative): This is a very rare species and only known from two subpopulations in Pico island (Amorim 2005). From all the samples taken, very few specimens were captured.

#### Subpopulations

Trend: Decline (inferred)

Justification for trend: In at least one of the subpopulations, the threats are sufficient for placing the subpopulations at risk of extinction (Gruta da Ribeira do Fundo).

Extreme fluctuations?: No

Severe fragmentation?: No

#### Habitat

System: Terrestrial

Habitat specialist: Yes

Habitat (narrative): This species occurs in two small lave tubes located on Pico island (Gruta das Cabras II and Gruta da Ribeira do Fundo) (Borges et al. 2004).

Trend in extent, area or quality?: Decline (inferred)

Justification for trend: Due to human activities involving garbage and solid waste dumping, as well as livestock farming, a loss of habitat quality is inferred.

##### Habitat

Habitat importance: Major Importance

Habitats: 7.1. Caves and Subterranean Habitats (non-aquatic) - Caves

#### Habitat

Habitat importance: Major Importance

Habitats: 7.1. Caves and Subterranean Habitats (non-aquatic) - Caves

#### Ecology

Size: 3.02–3.19 mm

Generation length (yr): 1

Dependency of single sp?: No

Ecology and traits (narrative): *Trechus pereirai* is a cavernicolous (i.e. a troglobitic species) predator and/or saprophagous species.

#### Threats

Justification for threats: This species is mainly threatened by human activities, especially garbage dumping and livestock farming affecting his habitat (Gruta da Ribeira do Fundo). However, there are several future potential threats: climatic changes will impact many habitats in the Azores (see Ferreira et al. 2016) and this can change the conditions inside the caves; change in the road infrastructure around the cave (Gruta das Cabras II); potential human recreational activities with disturbance caused by radical cave visitation; forest logging (Gruta das Cabras II).

##### Threats

Threat type: Ongoing

Threats: 1.1. Residential & commercial development - Housing & urban areas1.3. Residential & commercial development - Tourism & recreation areas2.1.2. Agriculture & aquaculture - Annual & perennial non-timber crops - Small-holder farming6.1. Human intrusions & disturbance - Recreational activities7.2.1. Natural system modifications - Dams & water management/use - Abstraction of surface water (domestic use)9.1.2. Pollution - Domestic & urban waste water - Run-off

##### Threats

Threat type: Future

Threats: 2.2.2. Agriculture & aquaculture - Wood & pulp plantations - Agro-industry plantations4.1. Transportation & service corridors - Roads & railroads11.1. Climate change & severe weather - Habitat shifting & alteration11.2. Climate change & severe weather - Droughts

#### Threats

Threat type: Ongoing

Threats: 1.1. Residential & commercial development - Housing & urban areas1.3. Residential & commercial development - Tourism & recreation areas2.1.2. Agriculture & aquaculture - Annual & perennial non-timber crops - Small-holder farming6.1. Human intrusions & disturbance - Recreational activities7.2.1. Natural system modifications - Dams & water management/use - Abstraction of surface water (domestic use)9.1.2. Pollution - Domestic & urban waste water - Run-off

#### Threats

Threat type: Future

Threats: 2.2.2. Agriculture & aquaculture - Wood & pulp plantations - Agro-industry plantations4.1. Transportation & service corridors - Roads & railroads11.1. Climate change & severe weather - Habitat shifting & alteration11.2. Climate change & severe weather - Droughts

#### Conservation

Justification for conservation actions: The species is protected by regional law (RAA 2012). As a future measure forconservation, it is suggested to fence the entrance of the caves where the species occurs. A habitat management plan is needed and is anticipated to be developed during the coming years.

##### Conservation actions

Conservation action type: In Place

Conservation actions: 1. Land/water protection2. Land/water management

##### Conservation actions

Conservation action type: Needed

Conservation actions: 2.1. Land/water management - Site/area management5.4.3. Law & policy - Compliance and enforcement - Sub-national level4.1. Education & awareness - Formal education

##### Conservation actions

Conservation action type: In Place

#### Conservation actions

Conservation action type: In Place

Conservation actions: 1. Land/water protection2. Land/water management

#### Conservation actions

Conservation action type: Needed

Conservation actions: 2.1. Land/water management - Site/area management5.4.3. Law & policy - Compliance and enforcement - Sub-national level4.1. Education & awareness - Formal education

#### Conservation actions

Conservation action type: In Place

#### Other

Justification for use and trade: The species is not utilised.

Justification for ecosystem services : Insufficient information available.

##### Use and trade

Use type: International

##### Ecosystem services

Ecosystem service type: Less important

##### Research needed

Research needed: 1.2. Research - Population size, distribution & trends1.3. Research - Life history & ecology2.2. Conservation Planning - Area-based Management Plan3.1. Monitoring - Population trends3.4. Monitoring - Habitat trends

Justification for research needed: Additional research is needed in order to determine the levels of population size as well as its ecology and life history. It is also necessary to establish a monitoring plan for the invertebrate community in the habitat in order to contribute to the conservation of this species.

#### Use and trade

Use type: International

#### Ecosystem services

Ecosystem service type: Less important

#### Research needed

Research needed: 1.2. Research - Population size, distribution & trends1.3. Research - Life history & ecology2.2. Conservation Planning - Area-based Management Plan3.1. Monitoring - Population trends3.4. Monitoring - Habitat trends

Justification for research needed: Additional research is needed in order to determine the levels of population size as well as its ecology and life history. It is also necessary to establish a monitoring plan for the invertebrate community in the habitat in order to contribute to the conservation of this species.

#### Viability analysis

### Trechus picoensis

#### Species information

Scientific name: Trechus picoensis

Species authority: Machado, 1988

Common names: Cave ground-beetle (English); Carocho cavernícola (Portuguese)

Kingdom: Animalia

Phylum: Arthropoda

Class: Insecta

Order: Coleoptera

Family: Carabidae

**Figure 10.**
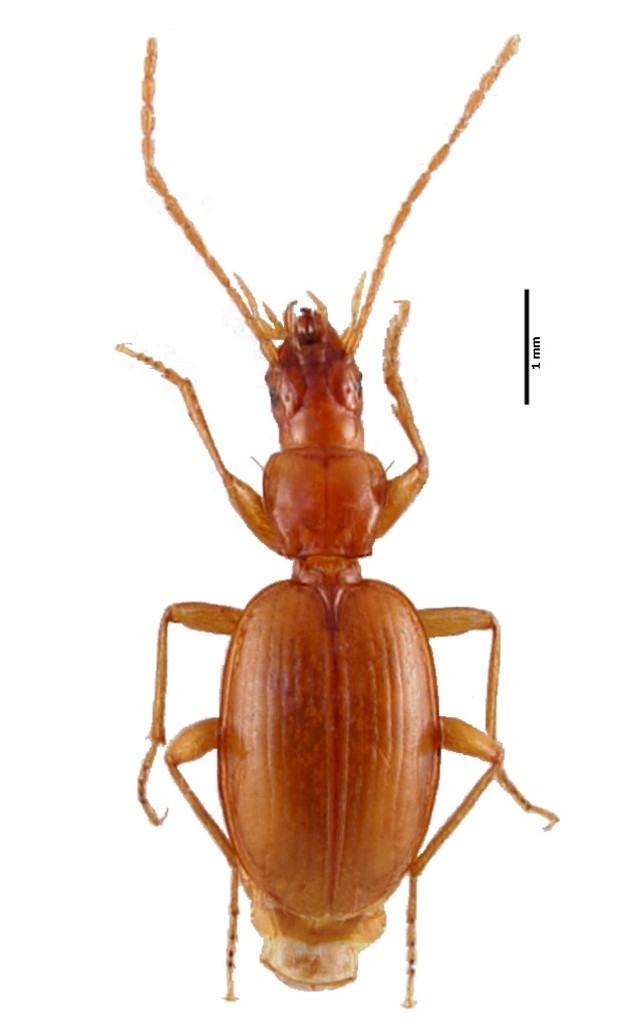
*Trechus picoensis* (Machado, 1988) from Pico island (Azores, Portugal) (Credit: Enésima Mendonça; Azorean Biodiversity Group, cE3c).

Figure(s) or Photo(s): Fig. 10

Region for assessment: Global

#### Editor & Reviewers

##### Reviewers

Reviewers: Anja Danielczak

##### Editor

Editor: Axel Hochkirch

#### Reviewers

Reviewers: Anja Danielczak

#### Editor

Editor: Axel Hochkirch

#### Geographic range

Biogeographic realm: Palearctic

Countries: Portugal

Map of records (Google Earth): Suppl. material 14

Basis of EOO and AOO: Observed

Basis (narrative): The extent of occurrence (EOO) is 285 km^2^ and the maximum estimated area of occupancy (AOO) is 40 km^2^.

Min Elevation/Depth (m): 10

Max Elevation/Depth (m): 770

Range description: *Trechus picoensis* is a cave-adapted endemic species known from Pico (Azores, Portugal) (Borges et al. 2010b). It is occurring in several lava tubes (Furna da Baliza, Furna de Frei Matias, Furna das Cabras II, Furna dos Montanheiros, Furna Nova I, Gruta do Gabriel, Gruta do Henrique Maciel, Gruta da Ribeira do Fundo and Gruta das Torres) (Pereira et al. 2015).

#### New occurrences

#### Extent of occurrence

EOO (km2): 285

Trend: Decline (observed)

Justification for trend: It has a relatively small extent of occurrence (EOO = 285 km²) and the area surrounding some of the caves is heavily impacted by human activities, namely agriculture/livestock farming and unregulated visitation to some of the caves.

Causes ceased?: No

Causes understood?: Yes

Causes reversible?: Unknown

Extreme fluctuations?: Unknown

#### Area of occupancy

Trend: Decline (observed)

Justification for trend: It has a reduced area of occupancy (AOO = 40 km²) and the area surrounding some of the caves is heavily impacted by human activities.

Causes ceased?: No

Causes understood?: Yes

Causes reversible?: Unknown

Extreme fluctuations?: Unknown

AOO (km2): 40

#### Locations

Number of locations: 9

Justification for number of locations: This species occurs in Pico island in nine lava-tubes (Furna da Baliza, Furna de Frei Matias, Furna das Cabras II, Furna dos Montanheiros, Furna Nova I, Gruta do Gabriel, Gruta do Henrique Maciel, Gruta da Ribeira do Fundo and Gruta das Torres) that are isolated in a sea of pastures and *Cryptomeria japonica* plantations and under several threats, namely the reduction of habitat quality.

Trend: Decline (inferred)

Justification for trend: Degradation of the habitat is observed due to human activities: agriculture/livestock farming and unregulated visitation to some of the caves.

Extreme fluctuations?: Unknown

#### Population

Number of individuals: Unknown

Trend: Decline (inferred)

Justification for trend: The decreasing abundance of some subpopulations is inferred from the decrease in habitat quality and pollution derived from dairy cattle exploitation.

Basis for decline: (c) a decline in area of occupancy, extent of occurrence and/or quality of habitat

Causes ceased?: No

Causes understood?: Yes

Causes reversible?: Unknown

Extreme fluctuations?: Unknown

Population Information (Narrative): The species is particularly abundant in some of the caves in Pico island.

#### Subpopulations

Trend: Decline (inferred)

Justification for trend: Some of the subpopulations are under intense threat due to pollution derived from dairy cattle exploitation.

Extreme fluctuations?: No

Severe fragmentation?: No

#### Habitat

System: Terrestrial

Habitat specialist: Yes

Habitat (narrative): This species occurs in several volcanic caves (lava-tubes) of Pico island. It shows a strong morphological adaptation to cave life (Machado 1988, Amorim 2005).

Trend in extent, area or quality?: Decline (inferred)

Justification for trend: The area surrounding some of the caves is heavily impacted by human activities: livestock farming/agriculture, timber production (*Cryptomeria japonica*) and by human intrusion and disturbance.

##### Habitat

Habitat importance: Major Importance

Habitats: 7.1. Caves and Subterranean Habitats (non-aquatic) - Caves

#### Habitat

Habitat importance: Major Importance

Habitats: 7.1. Caves and Subterranean Habitats (non-aquatic) - Caves

#### Ecology

Size: 4.5-5.4 mm

Generation length (yr): 1

Dependency of single sp?: No

Ecology and traits (narrative): *Trechus picoensis* is a cavernicolous (i.e. a troglobitic species) predator and/or saprophagous species.

#### Threats

Justification for threats: The main current threats to this species are the loss of habitat quality due to human activities: agriculture/livestock farming, forest logging and unregulated visitation to some of the caves. However, there are several future potential threats: climatic changes will impact many habitats in the Azores (see Ferreira et al. 2016) and this can change the conditions inside the caves; change in the road infrastructure around the caves; potential human recreational activities with disturbance caused by radical cave visitation.

##### Threats

Threat type: Ongoing

Threats: 2.1.2. Agriculture & aquaculture - Annual & perennial non-timber crops - Small-holder farming2.2.2. Agriculture & aquaculture - Wood & pulp plantations - Agro-industry plantations6.1. Human intrusions & disturbance - Recreational activities9.2.1. Pollution - Industrial & military effluents - Oil spills11.2. Climate change & severe weather - Droughts

##### Threats

Threat type: Future

Threats: 4.1. Transportation & service corridors - Roads & railroads11.1. Climate change & severe weather - Habitat shifting & alteration

#### Threats

Threat type: Ongoing

Threats: 2.1.2. Agriculture & aquaculture - Annual & perennial non-timber crops - Small-holder farming2.2.2. Agriculture & aquaculture - Wood & pulp plantations - Agro-industry plantations6.1. Human intrusions & disturbance - Recreational activities9.2.1. Pollution - Industrial & military effluents - Oil spills11.2. Climate change & severe weather - Droughts

#### Threats

Threat type: Future

Threats: 4.1. Transportation & service corridors - Roads & railroads11.1. Climate change & severe weather - Habitat shifting & alteration

#### Conservation

Justification for conservation actions: The species is not protected by regional law. Some of the caves are in a regionally protected area (Natural Park of Pico). Degraded habitats should be restored, a strategy needs to be developed to address the future threat by climate change that may change the vegetation cover above the caves and the entrance to the caves, most impacted by unregulated human visitation, should be fenced. A habitat management plan is needed and is anticipated to be developed during the coming years.

##### Conservation actions

Conservation action type: In Place

#### Conservation actions

Conservation action type: In Place

#### Other

Justification for use and trade: This species is not utilised

Justification for ecosystem services : Insufficient information available.

##### Use and trade

Use type: International

##### Ecosystem services

Ecosystem service type: Less important

##### Research needed

Research needed: 1.2. Research - Population size, distribution & trends1.3. Research - Life history & ecology2.2. Conservation Planning - Area-based Management Plan3.1. Monitoring - Population trends3.4. Monitoring - Habitat trends

Justification for research needed: Additional research is needed in order to determine the levels of population size, as well as its ecology and life history. It is also necessary to establish a monitoring plan for the invertebrate community in the habitat in order to contribute to the conservation of this species.

#### Use and trade

Use type: International

#### Ecosystem services

Ecosystem service type: Less important

#### Research needed

Research needed: 1.2. Research - Population size, distribution & trends1.3. Research - Life history & ecology2.2. Conservation Planning - Area-based Management Plan3.1. Monitoring - Population trends3.4. Monitoring - Habitat trends

Justification for research needed: Additional research is needed in order to determine the levels of population size, as well as its ecology and life history. It is also necessary to establish a monitoring plan for the invertebrate community in the habitat in order to contribute to the conservation of this species.

#### Viability analysis

### Trechus terceiranus

#### Species information

Scientific name: Trechus terceiranus

Species authority: Machado, 1988

Common names: Cave ground-beetle (English); Carocho cavernícola (Portuguese)

Kingdom: Animalia

Phylum: Arthropoda

Class: Insecta

Order: Coleoptera

Family: Carabidae

**Figure 11.**
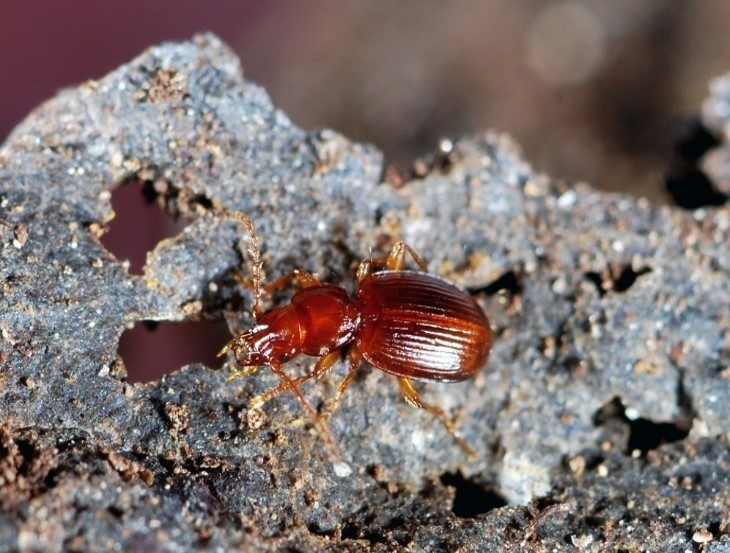
*Trechus terceiranus* (Machado, 1988) from Terceira island (Azores, Portugal) (Credit: Paulo A.V. Borges).

Figure(s) or Photo(s): Fig. 11

Region for assessment: Global

#### Editor & Reviewers

##### Reviewers

Reviewers: Anja Danielczak

##### Editor

Editor: Axel Hochkirch

#### Reviewers

Reviewers: Anja Danielczak

#### Editor

Editor: Axel Hochkirch

#### Geographic range

Biogeographic realm: Palearctic

Countries: Portugal

Map of records (Google Earth): Suppl. material 15

Basis of EOO and AOO: Observed

Basis (narrative): The extent of occurrence (EOO) is 48 km² and the maximum estimated area of occupancy (AOO) is 44 km².

Min Elevation/Depth (m): 250

Max Elevation/Depth (m): 583

Range description: *Trechus terceiranus* is a widespread cave-adapted endemic species from Terceira (Azores, Portugal) (Borges et al. 2010b), known from several caves (Algar do Carvão, Gruta da Achada, Gruta dos Balcões, Gruta do Caldeira, Gruta do Coelho, Gruta da Malha, Gruta do Natal, Gruta dos Principiantes, Gruta do Chocolate and Gruta de Santa Maria). The species was also found in the MSS (”Milieu Souterrain Superficiel“ or ”Mesovoid Shallow Substratum“) (Borges 1993) in the area of Pico Rachado, far from the location of known caves.

#### New occurrences

#### Extent of occurrence

EOO (km2): 48

Trend: Stable

Justification for trend: It has an extent of occurrence (EOO) of 48 km² and the species is known from ten isolated subpopulations, but also occurs in "Milieu Souterrain Superficiel“ or ”Mesovoid Shallow Substratum" (MSS).

Causes ceased?: Unknown

Causes understood?: Yes

Causes reversible?: Unknown

Extreme fluctuations?: Unknown

#### Area of occupancy

Trend: Stable

Justification for trend: It has a reduced area of occupancy (AOO = 44 km²), but is common in many caves.

Causes ceased?: Unknown

Causes understood?: Yes

Causes reversible?: Unknown

Extreme fluctuations?: Unknown

AOO (km2): 44

#### Locations

Number of locations: 10

Justification for number of locations: The species occurs in ten volcanic pits and/or lava-tubes (Algar do Carvão, Gruta da Achada, Gruta dos Balcões, Gruta do Caldeira, Gruta do Coelho, Gruta da Malha, Gruta do Natal, Gruta dos Principiantes, Gruta do Chocolate and Gruta de Santa Maria), that are under several potential threats.

Trend: Unknown

Extreme fluctuations?: No

#### Population

Number of individuals: Unknown

Trend: Stable

Justification for trend: The species is known from ten isolated abundant subpopulations, but the area surrounding some of the caves is not protected and we assume some possible future impacts for those isolated subpopulations. It is a widespread cave-adapted endemic species from Terceira (Azores, Portugal).

Causes ceased?: No

Causes understood?: Yes

Causes reversible?: Unknown

Extreme fluctuations?: No

Population Information (Narrative): The species is particularly abundant in Terceira island.

#### Subpopulations

Trend: Stable

Justification for trend: The species is known from ten isolated abundant subpopulations, but the area surrounding some of the caves is not protected and we assume some possible future impacts for those isolated subpopulations.

Extreme fluctuations?: No

Severe fragmentation?: No

#### Habitat

System: Terrestrial

Habitat specialist: Yes

Habitat (narrative): This species occurs in several volcanic formations (lava tubes and volcanic pits) of Terceira island. This species is distributed by all hypogean environments of Terceira both in the cave and MSS - mesocavernous shallow stratum habitats (Borges 1993, Amorim 2005).

Trend in extent, area or quality?: Decline (observed)

Justification for trend: The area surrounding some of the caves is heavily impacted by human activities and, in the Algar do Carvão Show-Cave, there is an ongoing impact from tourism visitation.

##### Habitat

Habitat importance: Major Importance

Habitats: 7.1. Caves and Subterranean Habitats (non-aquatic) - Caves

#### Habitat

Habitat importance: Major Importance

Habitats: 7.1. Caves and Subterranean Habitats (non-aquatic) - Caves

#### Ecology

Size: 3.6-4.3 mm

Generation length (yr): 1

Dependency of single sp?: No

Ecology and traits (narrative): *Trechus terceiranus* is a cavernicolous (i.e. a troglobitic species) predator and/or saprophagous species. Based on monthly data collected from the Algar do Carvão show cave during ten years, we can confirm that this species is active all months of the year, but with a high density between May and September.

#### Threats

Justification for threats: The main current threat to this species is cave visitation by tourists and the impact of agriculture activities. However, there are several future potential threats: climatic changes will impact many habitats in the Azores (see Ferreira et al. 2016) and this can change the conditions inside the caves; change in the road infrastructure around the caves; potential human recreational activities with disturbance caused by radical cave visitation.

##### Threats

Threat type: Ongoing

Threats: 2.1.2. Agriculture & aquaculture - Annual & perennial non-timber crops - Small-holder farming2.2.2. Agriculture & aquaculture - Wood & pulp plantations - Agro-industry plantations6.1. Human intrusions & disturbance - Recreational activities

##### Threats

Threat type: Future

Threats: 4.1. Transportation & service corridors - Roads & railroads11.1. Climate change & severe weather - Habitat shifting & alteration11.2. Climate change & severe weather - Droughts

#### Threats

Threat type: Ongoing

Threats: 2.1.2. Agriculture & aquaculture - Annual & perennial non-timber crops - Small-holder farming2.2.2. Agriculture & aquaculture - Wood & pulp plantations - Agro-industry plantations6.1. Human intrusions & disturbance - Recreational activities

#### Threats

Threat type: Future

Threats: 4.1. Transportation & service corridors - Roads & railroads11.1. Climate change & severe weather - Habitat shifting & alteration11.2. Climate change & severe weather - Droughts

#### Conservation

Justification for conservation actions: The species is not protected by regional law. Part of its habitat is in a regionally protected area (Natural Park of Terceira). Degraded habitats should be restored and a strategy needs to be developed to address the future threat by climate change. A habitat management plan is needed and is anticipated to be developed during the coming years.

##### Conservation actions

Conservation action type: In Place

Conservation actions: 1. Land/water protection2. Land/water management

##### Conservation actions

Conservation action type: Needed

Conservation actions: 2.1. Land/water management - Site/area management5.4. Law & policy - Compliance and enforcement4.1. Education & awareness - Formal education

#### Conservation actions

Conservation action type: In Place

Conservation actions: 1. Land/water protection2. Land/water management

#### Conservation actions

Conservation action type: Needed

Conservation actions: 2.1. Land/water management - Site/area management5.4. Law & policy - Compliance and enforcement4.1. Education & awareness - Formal education

#### Other

Justification for use and trade: The species is not utilised.

Justification for ecosystem services : Insufficient information available.

##### Use and trade

Use type: International

##### Ecosystem services

Ecosystem service type: Less important

##### Research needed

Research needed: 1.2. Research - Population size, distribution & trends1.3. Research - Life history & ecology2.2. Conservation Planning - Area-based Management Plan3.1. Monitoring - Population trends3.4. Monitoring - Habitat trends

Justification for research needed: Further research is needed into its ecology and life history in order to find extant specimens. It is necessary to establish a monitoring plan for the invertebrate community in the habitat in order to contribute to the conservation of this species.

#### Use and trade

Use type: International

#### Ecosystem services

Ecosystem service type: Less important

#### Research needed

Research needed: 1.2. Research - Population size, distribution & trends1.3. Research - Life history & ecology2.2. Conservation Planning - Area-based Management Plan3.1. Monitoring - Population trends3.4. Monitoring - Habitat trends

Justification for research needed: Further research is needed into its ecology and life history in order to find extant specimens. It is necessary to establish a monitoring plan for the invertebrate community in the habitat in order to contribute to the conservation of this species.

#### Viability analysis

## Discussion

In this study, we have studied 15 Azorean endemic arthropods with some level of cave adaptation, grouping the historical data and giving new information about its distribution, habitat, threats and proposals for its conservation. Only three out of the 15 studied species are known from more than one island and the remaining are restricted to single islands. The three more widespread species are one Collembola, one pseudoscorpion and a spider and all of them may need some level of taxomic revision using molecular tools.

However, most of the Azorean cave arthropod species have a very restricted distribution, occupying a unique island and most of the times few caves and therefore they have a very small extent of occurrence (EOO) and area of occupancy (AOO). The lack of new records makes us consider the possibility that one of the species is extinct (*Cixius cavazoricus*). In fact, the two troglobitic species of the homopteran genus *Cixius* are under intense disturbance due to major land-use changes in epigean habitats above their known localities. In addition, many others species are in a critical conservation situation and actions should be taken with some urgency, namely the implementation of area-based management plans for the specifc caves.

Borges et al. 2012 evaluated the status of Azorean lava-tubes and volcanic pits concerning species diversity and rarity, concluding that, to preserve all troglobiont arthropods endemic to the Azores, it is crucial to protect several caves per island. Since then, there has been an effort to create a new law for the protection of the most important Azorean caves and is expected to be published in 2019-2020. However, many caves in Terceira, Faial and Pico Islands are being impacted by pollution due to the intensive cattle production, with the cha,ges in ecological conditions of caves in the last 50 years, namely the change in the nitrogen and phosphorous abiotic cycles and changes in the water pH (see Hathaway et al. 2014). Therefore, there is some urgency to perform new standardised surveys in the most important caves from the Azores to understand the current status of populations of the 15 species.

**Figure 12.**
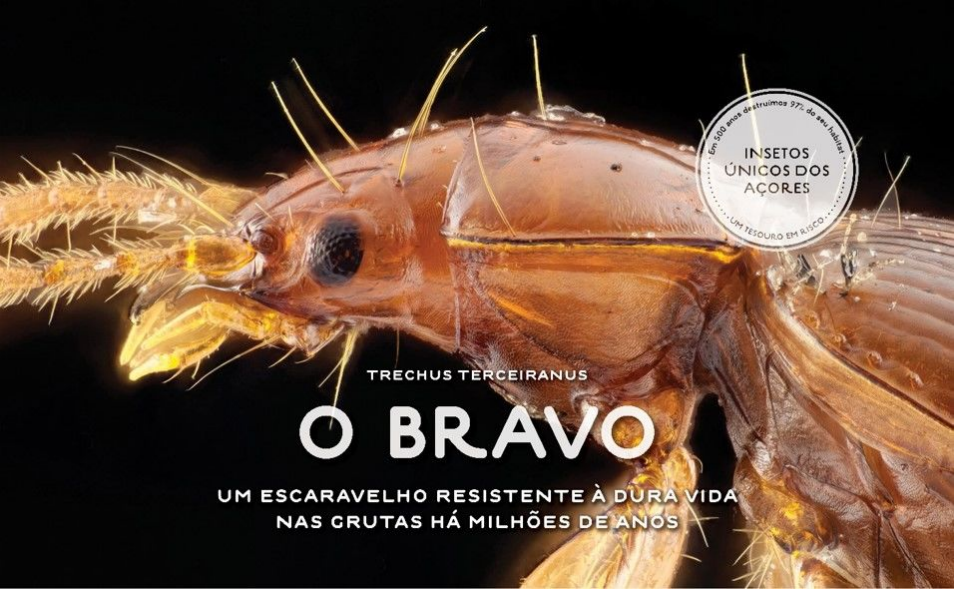
*Trechus terceiranus* extreme macro photo used for an urban outreach initiative (Credit: Javier Torrent, Azorean Biodiversity Group, cE3c).

Formal education and awareness is needed to allow future investments in cave protection that will create conflicts with farmers. The use of images from extreme macro can be a strategy to inform the public about the ecological an aesthetic value of Azorean endemic cave arthropods as was demonstrated by the recent urban outreach initiative at Angra do Heroísmo in Terceira Island (see Amorim et al. 2016) (Fig. 12).

Discussion

## Supplementary Material

Supplementary material 1 Macarorchestia martini
Data type: Map Google Earth
Brief description: Distribution of *Macarorchestia martini* in the Azores islands
File: oo_248839.kmz Anja Danielczak

Supplementary material 2 *Pseudosinella ashmoleorum*
Data type: Map Google Earth
Brief description: Distribution of *Pseudosinella ashmoleorum* in Azores islands.
File: oo_248841.kmz Anja Danielczak

Supplementary material 3 *Pseudoblothrus oromii*
Data type: Map Google Earth
Brief description: Distribution of *Pseudoblothrus oromii* in Azores islands.
File: oo_248842.kmz Anja Danielczak

Supplementary material 4 *Pseudoblothrus vulcanus*
Data type: Map Google Earth
Brief description: Distribution of *Pseudoblothrus vulcanus* in Azores islands.
File: oo_248843.kmz Anja Danielczak

Supplementary material 5 *Rugathodes pico*
Data type: Map Google Earth
Brief description: Distribution of *Rugathodes pico* in Azores islands.
File: oo_248844.kmz Anja Danielczak

Supplementary material 6 *Cixius cavazoricus*
Data type: Map Google Earth
Brief description: Distribution of *Cixius cavazoricus* in Azores islands.
File: oo_248845.kmz Anja Danielczak

Supplementary material 7 *Cixius azopicavus*
Data type: Map Google Earth
Brief description: Distribution of *Cixius azopicavus* in Azores islands.
File: oo_248846.kmz Anja Danielczak

Supplementary material 8 *Thalassophilus azoricus*
Data type: Map Google Earth
Brief description: Distribution of *Thalassophilus azoricus* in Azores islands.
File: oo_248847.kmz Anja Danielczak

Supplementary material 9 *Trechus isabelae*
Data type: Map Google Earth
Brief description: Distribution of *Trechus isabelae* in Azores.
File: oo_248849.kmz Anja Danielczak

Supplementary material 10 *Trechus jorgensis*
Data type: Map Google Earth
Brief description: Distribution of *Trechus jorgensis* in Azores
File: oo_248852.kmz Anja Danielczak

Supplementary material 11 *Trechus montanheirorum*
Data type: Map Google Earth
Brief description: Distribution of *Trechus montanheirorum* in Azores.
File: oo_248854.kmz Anja Danielczak

Supplementary material 12 *Trechus oromii*
Data type: Map Google Earth
Brief description: Distribution of *Trechus oromii* in Azores.
File: oo_248856.kmz Anja Danielczak

Supplementary material 13 *Trechus pereirai*
Data type: Map Google Earth
Brief description: Distribution of *Trechus pereirai* in Azores.
File: oo_248860.kmz Anja Danielczak

Supplementary material 14 *Trechus picoensis*
Data type: Map Google Earth
Brief description: Distribution of *Trechus picoensis* in Azores.
File: oo_248864.kmz Anja Danielczak

Supplementary material 15 *Trechus terceiranus*
Data type: Map Google Earth
Brief description: Distribution of *Trechus terceiranus* in Azores.
File: oo_248869.kmz Anja Danielczak
